# Stochastic growth and ligand–receptor interaction-mediated stabilization generate stereotyped dendritic arbors

**DOI:** 10.1038/s41593-026-02278-0

**Published:** 2026-05-04

**Authors:** Rebecca Shi, Xue Yan Ho, Li Tao, Landon Bayless-Edwards, Caitlin A. Taylor, Ting Zhao, Wei Zou, Malcolm Lizzappi, Kelsie Eichel, Tianyi Mao, Kang Shen

**Affiliations:** 1 Department of Biology, Stanford University, Stanford, CA USA; 2 Neurosciences IDP Program, Stanford University, Stanford, CA USA; 3 Howard Hughes Medical Institute, Stanford University, Stanford, CA USA; 4 Vollum Institute, Oregon Health & Science University, Portland, OR USA; 5 Knight Initiative of Brain Resilience, Stanford University, Stanford, CA USA; 6 The Fourth Affiliated Hospital, Zhejiang University School of Medicine, Yiwu, China; 7 Institute of Translational Medicine, Zhejiang University, Hangzhou, China

**Keywords:** Development of the nervous system, Developmental biology, Axon and dendritic guidance

## Abstract

Stereotyped dendritic arbors are shaped by dynamic and stochastic growth during neuronal development. It remains unclear how guidance receptors and ligands coordinate branch dynamic growth, retraction and stabilization to specify dendritic arbors. We previously showed that extracellular adhesion ligand SAX-7/LICAM dictates the elaborate and stereotyped shape of the *Caenorhabditis elegans* PVD sensory dendrite via binding to the guidance receptor DMA-1, a single transmembrane adhesion molecule. Here, we perform structure–function analyses of DMA-1 and unexpectedly find that robust, stochastic dendritic growth does not require ligand binding. Instead, ligand contacts prevent dendrite retraction, inhibit ectopic growth and specify arbor shape. Furthermore, we demonstrate that dendritic growth requires a pool of ligand-free DMA-1, which is maintained by receptor endocytosis and reinsertion to the plasma membrane via recycling endosomes. Mutants defective of DMA-1 endocytosis show severely truncated dendrites. We present a model in which ligand-free guidance receptor mediates intrinsic, stochastic dendritic growth, while extracellular ligands instruct dendrite shape by inhibiting growth.

## Abstract (web summary)

Stereotyped dendritic arbors arise from stochastic growth and selective stabilization of dendritic branches. Two pools of guidance receptor are required. Ligand-free receptors drive stochastic growth while ligand-bound receptors stabilize branches.

## Main

The size and shape of dendritic arbors are determined by cell-intrinsic mechanisms including transcription factors and cytoskeletal regulators^1,2^, cell–cell or cell–matrix interactions (guidance cues and receptors)^3^ and activity-dependent mechanisms^4,5^. Dendritic development, as revealed by imaging studies, is a dynamic and stochastic process. Individual developing dendrites cycle through dendritic initiation, growth and retraction to establish the shape of dendrites in many species^3,6,7^. Indeed, computational models can predict dendritic shape based on the dynamic features of branch tips and the rapid transitions between growth and retraction^8^. Molecularly, the prevailing model for neuronal morphogenesis is that extrinsic cues activate cell surface receptors which in turn regulate cytoskeleton re-organization^9^. However, we do not understand how guidance receptors and their extracellular ligands mediate these complex and dynamic growth processes to build mature neurons with stereotyped arbors.

We studied the *Caenorhabditis elegans* sensory neuron PVD, which has a large and branched dendrite arbor^10^. The shape of PVD dendrites is determined by three extracellular cues (SAX-7, MNR-1, LECT-2) expressed by target tissues and by a receptor complex (DMA-1 and HPO-30) on the dendrite. Among the ligands, SAX-7, the ortholog for the vertebrate L1CAM, is a transmembrane protein on skin cells and localizes at regularly spaced intervals^3,11,12^. The activation of DMA-1 requires all three ligands to specify the growth and branching of PVD dendrites in the extracellular space between the skin and muscle^3^. In PVD, DMA-1 binds to its co-receptor, a claudin homolog HPO-30, which is also required for dendritic growth^13^. This DMA-1–HPO-30 co-receptor complex recruits actin regulators, including TIAM-1 and the WAVE regulatory complex, to promote actin polymerization and dendritic growth^13–15^. Additionally, the furin-like proprotein convertase KPC-1 functions in PVD to promote dendritic growth^16–18^. KPC-1 downregulates overall protein levels of DMA-1^16,17^. However, it is unclear how KPC-1 regulates dendritic growth through its convertase activity and why increased levels of DMA-1 in *kpc-1(null)* lead to severe loss of PVD dendrites.

## Results

### Ligand-free DMA-1 receptor supports dendritic growth

**Fig. 1:**
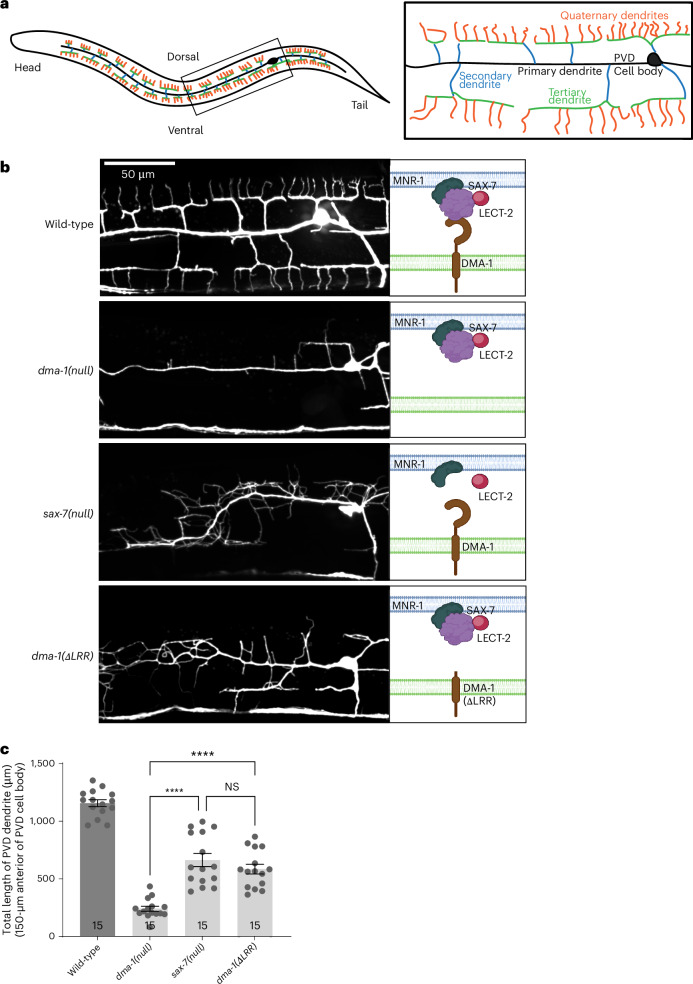
Ligand-free DMA-1 receptor supports dendritic growth. **a**, Schematic of a wild-type animal with PVD neuron (left) and close-up of the boxed region (right). Anterior is left and ventral is down in this and all following images showing PVD morphology. **b**, Representative images of PVD dendrite morphology in wild-type, *dma-1(null)*, *sax-7(null)* and *dma-1(ΔLRR)* mutant animals at 1-day-old adult stage (left). Scale bar: 50 µm. Image representative of 15 animals. Right: schematic images of molecules that are present in the corresponding animals. **c**, Quantification of the total length of PVD dendrite, 150 µm anterior to the PVD cell body. Data are presented as mean values ± s.e.m. *n* values within each bar. Statistical comparison was performed using Brown–Forsythe one-way ANOVA with Dunnett’s multiple comparisons test. *P* < 0.001 (*dma-1(null)* versus *sax-7(null)*), *P* < 0.001 (*dma-1(null)* versus *dma-1(ΔLRR)*), *P* = 0.8350 (*sax-7(null)* versus *dma-1(ΔLRR)*). ****P < 0.0001. NS, not significant. Schematic in **b** created in BioRender; Ho, X. Y. https://biorender.com/kx803o4 (2026).

The PVD dendrites consist of 1°, 2°, 3° and 4° dendritic branches, resulting in numerous menorah-like structures (Fig. 1a,b). *dma-1(null)* mutants show dramatically reduced total dendritic length and completely lack menorah (Fig. 1b,c). Interestingly, *sax-7* mutants do not phenocopy *dma-1(null)* mutants. While *sax-7(null)* mutants also lack menorahs, the total dendritic length is consistently more extensive than *dma-1(null)* mutants. The disorganized arbors observed in *sax-7* animals require DMA-1 because *dma-1; sax-7* double mutants phenocopy *dma-1* mutants^3^. This indicates that DMA-1 can promote disorganized dendrite growth in the absence of SAX-7. We predict that DMA-1 plays distinct roles to promote growth (independent of SAX-7) and stabilization (dependent on SAX-7).

DMA-1 is a single transmembrane protein with an extracellular domain, a transmembrane and a short intracellular region. The extracellular domain is predicted to contain a continuous leucine-rich-repeat domain (436 amino acids) and a 72-amino-acid juxtamembrane sequence. We used CRISPR–Cas9 to precisely delete the entire leucine-rich-repeat domain of DMA-1 from its endogenous locus (*dma-1(*Δ*LRR)*). This truncated DMA-1 molecule retains the juxtamembrane sequence, as well as intact transmembrane and intracellular domains. Interestingly, *dma-1(*Δ*LRR)* mutants displayed exuberant but disorganized arbors, indistinguishable from the *sax-7(null)* mutants, which is different from the severe loss of dendrites in the *dma-1(null)* (Fig. 1b,c). Together, these results suggest that even though the extracellular, ligand-binding domain of DMA-1 is required for establishing the arbor shape, the non-ligand-binding transmembrane and cytosolic domains of DMA-1 are sufficient to promote the growth and branching of dendrites.

### Ligand-bound DMA-1 receptor stabilizes dendritic branches

**Fig. 2:**
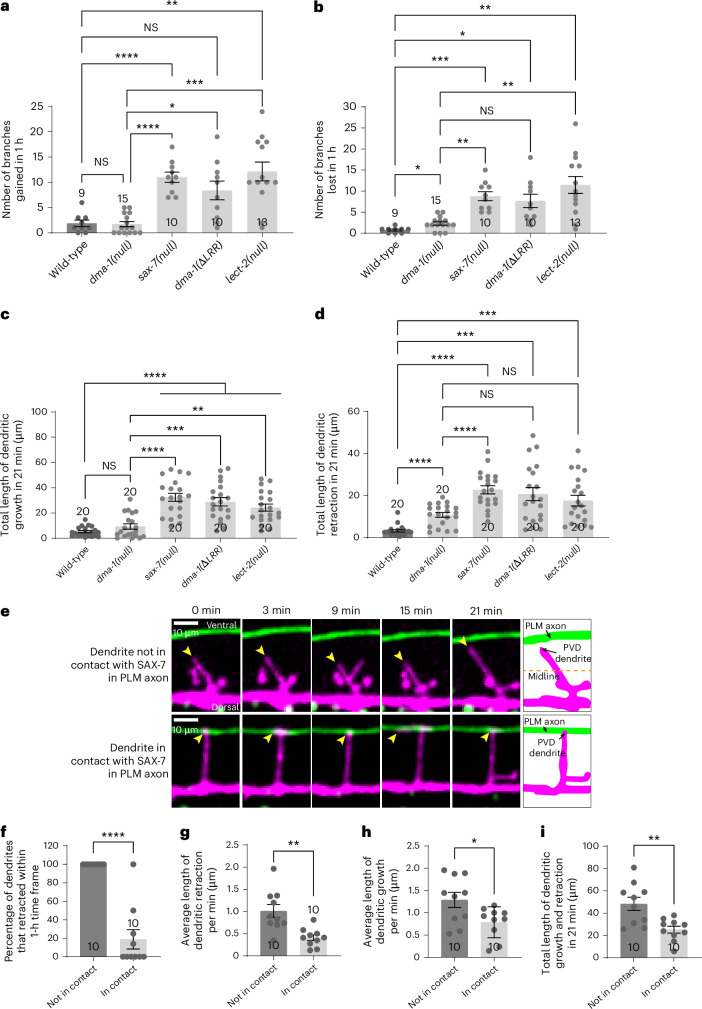
Ligand-bound DMA-1 receptor inhibits dendritic growth. **a**, Quantification of the number of branches gained. *P* > 0.9999 (wild-type versus *dma-1(null)*), *P* < 0.0001 (wild-type versus *sax-7(null)*), *P* = 0.9999 (wild-type versus *dma-1(ΔLRR)*), *P* = 0.6094 (wild-type versus *lect-2(null)*), *P* < 0.0001 (*dma-1(null)* versus *sax-7(null)*), *P* = 0.0491 (*dma-1(null)* versus *dma-1(ΔLRR)*), *P* = 0.0038 (*dma-1(null)* versus *lect-2(null)*). **b**, Quantification of the number of branches lost. *P* = 0.0308 (wild-type versus *dma-1(null)*), *P* = 0.0002 (wild-type versus *sax-7(null)*), *P* = 0.0162 (wild-type versus *dma-1(ΔLRR)*), *P* = 0.0018 (wild-type versus *lect-2(null)*), *P* = 0.001 (*dma-1(null)* versus *sax-7(null)*), *P* = 0.00675 (*dma-1(null)* versus *dma-1(ΔLRR)*), *P* = 0.0062 (*dma-1(null)* versus *lect-2(null)*). **c**, Quantification of total length of dendritic growth. *P* = 0.533 (wild-type versus *dma-1(null)*), *P* < 0.0001 (wild-type versus *sax-7(null)*), *P* < 0.0001 (wild-type versus *dma-1(ΔLRR)*), *P* < 0.0001 (wild-type versus *lect-2(null)*), *P* < 0.0001 (*dma-1(null)* versus *sax-7(null)*), *P* = 0.0002 (*dma-1(null)* versus *dma-1(ΔLRR)*), *P* = 0.0013 (*dma-1(null)* versus *lect-2(null)*). **d**, Quantification of total length of dendritic retraction. *P* < 0.0001 (wild-type versus *dma-1(null)*), *P* < 0.0001 (wild-type versus *sax-7(null)*), *P* = 0.0002 (wild-type versus *dma-1(ΔLRR)*), *P* = 0.0002 (wild-type versus *lect-2(null)*), *P* < 0.0001 (*dma-1(null)* versus *sax-7(null)*), *P* = 0.0574 (*dma-1(null)* versus *dma-1(ΔLRR)*), *P* = 0.1843 (*dma-1(null)* versus *lect-2(null)*). For **a**–**d**, animals are at L4 stage. Data are presented as mean ± s.e.m. *n* values within each bar. Statistical comparison was performed using Brown–Forsythe one-way ANOVA with Dunnett’s multiple comparisons test. **e**, Time-lapse imaging of developing PVD dendrites (magenta) and SAX-7s::YFP in PLM axons (green) in *Pmec-17::sax-7s::YFP; Pmec-17::mnr-1; ser2prom3::myr-mCherry; sax-7(null)* mutant animals. Top: representative image of a growing dendrite that was never in contact with the PLM axon. Bottom: representative image of a growing dendrite that was always in contact with the PLM axon. Yellow arrowheads indicate the tips of dendrites growing from the primary dendrite and PLM axon. Scale bar: 10 µm. Right: schematic images of growing dendrites at 21-min time point of corresponding conditions. Dashed lines indicate the midline used to quantify the percentage of dendrites that retracted. **f**, Quantification of the percentage of dendrites that retracted within 1 h for dendrites that were in contact with SAX-7, and for dendrites that were not in contact with SAX-7, *P* < 0.0001. **g**, Quantification of the average length of dendritic retraction per minute for both dendrites in contact with SAX-7 and dendrites not in contact with SAX-7, within 21 min, *P* = 0.0024. **h**, Quantification of the average length of dendritic growth per minute for both dendrites in contact with SAX-7 and dendrites not in contact with SAX-7, within 21 min, *P* = 0.0252. **i**, Quantification of total dendritic growth and retraction per minute for both dendrites in contact with SAX-7 and dendrites not in contact with SAX-7, *P* = 0.0039. This was calculated as the sum of both total growth and retraction over 21 min and measures the total movement of the tip of the dendrite. For **f**–**h**, data are presented as mean ± s.e.m. *n* values within each bar. Statistical comparisons were performed using Welsh’s unpaired two-way *t*-test. *P < 0.05, **P < 0.01, ***P < 0.001, ****P < 0.0001.

**Extended Data Fig. 1:**
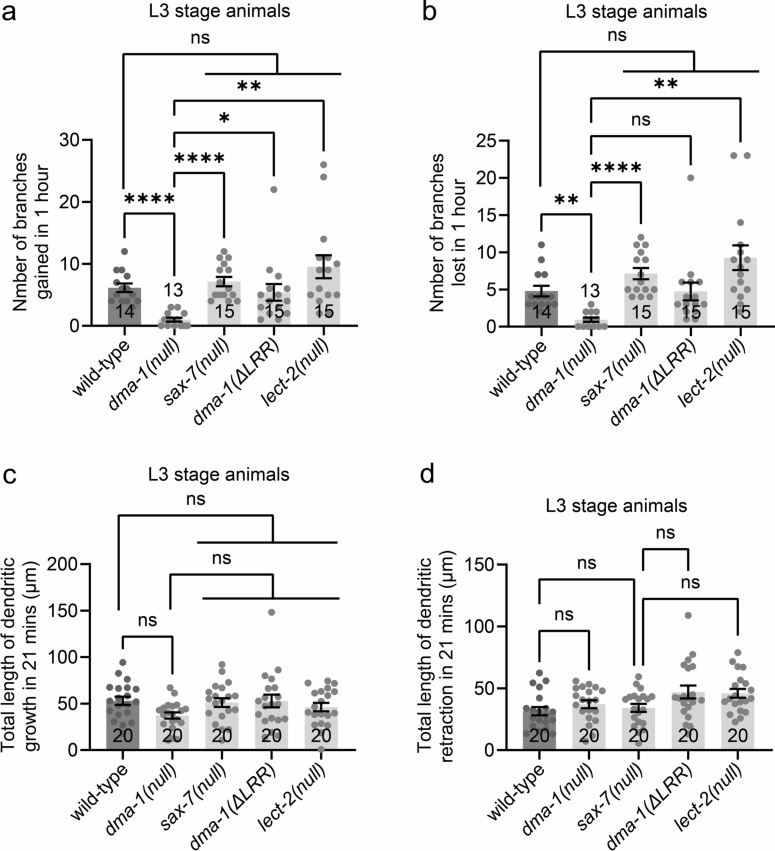
Ligand-bound DMA-1 receptor inhibits dendritic growth. (**a**) Quantification of number of branches gained. *P* < 0.0001 (wild-type versus *dma-1(null)*), *P* = 0.9762 (wild-type versus *sax-7(null)*), *P* = 0.9999 (wild-type versus *dma-1(ΔLRR)*), *P* = 0.6094 (wild-type versus *lect-2(null)*), *P* < 0.0001 (*dma-1(null)* versus *sax-7(null)*), *P* = 0.0491 (*dma-1(null)* versus *dma-1(ΔLRR)*), P = 0.0038 (*dma-1(null)* versus *lect-2(null)*). (**b**) Quantification of number of branches lost. *P* = 0.0010 (wild-type versus *dma-1(null)*), *P* = 0.2538 (wild-type versus *sax-7(null)*), *P* > 0.9999 (wild-type versus *dma-1(ΔLRR)*), *P* = 0.1847 (wild-type versus *lect-2(null)*), *P* < 0.0001 (*dma-1(null)* versus *sax-7(null)*), *P* = 0.0603 (*dma-1(null)* versus *dma-1(ΔLRR)*), P = 0.0016 (*dma-1(null)* versus *lect-2(null)*). (**c**) Total length of dendritic growth. *P* = 0.1354 (wild-type versus *dma-1(null)*), *P* > 0.9999 (wild-type versus *sax-7(null)*), *P* > 0.9999 (wild-type versus *dma-1(ΔLRR)*), *P* = 0.998 (wild-type versus *lect-2(null)*), *P* = 0.3360 (*dma-1(null)* versus *sax-7(null)*), *P* = 0.6067 (*dma-1(null)* versus *dma-1(ΔLRR)*), P = 0.8892 (*dma-1(null)* versus *lect-2(null)*). (**d**) Total length of dendritic retraction. *P* = 0.9672 (wild-type versus *dma-1(null)*), *P* > 0.9999 (wild-type versus *sax-7(null)*), *P* = 0.2199 (wild-type versus *dma-1(ΔLRR)*), *P* = 00715 (wild-type versus *lect-2(null)*), *P* > 0.9999 (*dma-1(null)* versus *sax-7(null)*), *P* = 0.8 (*dma-1(null)* versus *dma-1(ΔLRR)*), P = 0.6279 (*dma-1(null)* versus *lect-2(null)*). Data are presented as mean values +/− SEM. n values within each bar. Statistical comparison was performed using Brown-Forsythe one-way ANOVA with Dunnett’s multiple comparisons test. *P < 0.05, **P < 0.01, ****P < 0.0001.

Previous studies showed that developing dendrites are highly dynamic and undergo growth and retraction, while fully established dendrites are stabilized and much less dynamic^6^. Using time-lapse imaging, we quantified the numbers of PVD dendrite branch addition and retraction in different developmental stages. During the L3 stage, wild-type PVD exhibits robust branch addition, accompanied by branch retraction (Extended Data Fig. 1a–d and Supplementary Video 1). In contrast, at the end of the L4 stage when the PVD arbor is fully developed, dendrites show little continuous growth and retraction, thus exhibiting the typical transition from developmental dynamicity to stability (Fig. 2a–d and Supplementary Video 2).

To understand what underlies this transition, we imaged the growth and retraction of dendrites in ligand and receptor mutant animals. Consistent with previous reports, *dma-1(null)* showed little branch addition in the L3 stage, indicating that DMA-1 is required for branch growth^3^ (Extended Data Fig. 1a). Although numerous filopodia emerge from the primary dendrite of *dma-1(null)* mutants, they completely retract, leading to no net growth (Extended Data Fig. 1c,d and Supplementary Video 3). This result also suggests that different molecular mechanisms may underlie branch addition and filopodia extension.

Next, we tested whether ligand–receptor interaction is required for branch addition and stabilization by imaging ligand mutants—*sax-7(null)*, *lect-2(null)* and *dma-1(*Δ*LRR)* mutants. We observed that dendrites in *sax-7(null)*, *dma-1(*Δ*LRR)* and *lect-2(null)* mutants are different from *dma-1(null)*. The ligand mutants exhibit robust growth and retraction in the L3 stage (Extended Data Fig. 1a–d and Supplementary Videos 4–6), indicating that ligand binding is not required for branch addition. Interestingly, different from wild-type or *dma-1(null)*, the dynamic dendrite branching and retraction continued in the L4 stage in *sax-7(null)*, *dma-1(*Δ*LRR)* and *lect-2(null)* mutants, suggesting that ligand–receptor interaction is required for the dendrite stabilization (Fig. 2a–d and Supplementary Videos 7–10). These results indicate that ligand binding is required for the transition between the dynamic growth behavior and stable dendritic arbors and further support the notion that the intracellular domain of DMA-1 is sufficient to promote growth. Similar results were obtained from a *dma-1* allele containing three point mutations which eliminated ligand binding^19^.

To further determine whether ligand binding inhibits continuous growth and retraction, we examined the behavior of dendrites that do or do not contact SAX-7 during dendritic growth. The DMA-1 ligands SAX-7 and MNR-1 are widely expressed and broadly distributed across all levels of PVD branches in wild-type animals. Therefore, to observe the behavior of dendrites upon contact with SAX-7 and MNR-1, we spatially restricted the expression of SAX-7 and MNR-1 in mechanosensory PLM neurons, by expressing SAX-7 and MNR-1 cell-specifically in the mechanosensory neurons (*Pmec-17::sax-7s::YFP; Pmec-17::mnr-1*) in *sax-7(null)* mutant animals (Fig. 2e–i), and recorded the dynamic behavior of secondary dendrites that do or do not contact PLM. As expected, secondary dendrites that are not in contact with PLM exhibit growth and retraction along the dorsal ventral axis similar to *sax-7(null)* mutant animals (Fig. 2e). Upon contacting the SAX-7- and MNR-1-expressing PLM, developing dendrites stop growing along the dorsal ventral direction and start growing along the PLM axon (Fig. 2e and Supplementary Video 11). Quantitatively, 100% of dendrites not contacting SAX-7 and MNR-1 showed retraction events within an hour, while only a small percentage of dendrites contacting SAX-7 and MNR-1 showed retraction events (Fig. 2f). We also observed that the total retraction length is significantly shorter in dendrites that contact SAX-7 and MNR-1 compared with those that do not make contact (Fig. 2g). In contrast, the growth length was significantly longer in dendrites without contact, indicating that ligand interaction also inhibits the growth of secondary dendrites (Fig. 2h). Finally, we summed the total growth and retraction of dendritic tips as an indicator of dynamicity (Fig. 2i) and saw a reduction in dendrites that contacted SAX-7 and MNR-1 compared with dendrites that did not contact SAX-7 and MNR-1. Taken together, these results suggest that ligand binding to DMA-1 stabilizes the developing dendrite by preventing continuous dynamicity.

### The LRR of DMA-1 is dispensable for dendrite outgrowth

**Fig. 3:**
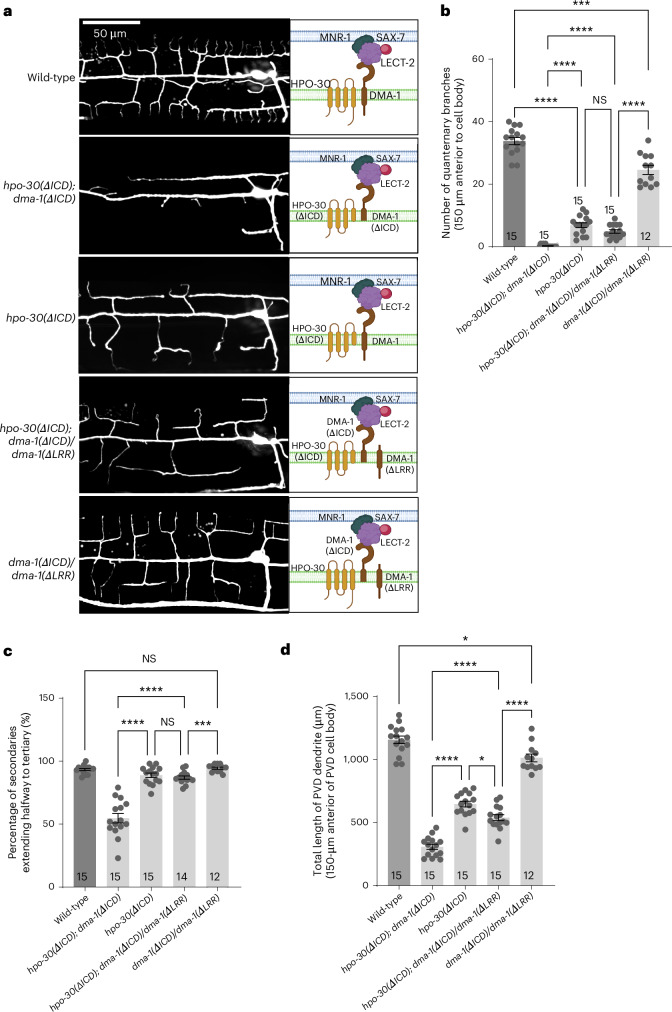
DMA-1 can function as two separate halves. **a**, Representative images of PVD dendrite morphology in wild-type, *hpo-30(ΔICD); dma-1(ΔICD)*, *hpo-30(ΔICD)*, *hpo-30(ΔICD); dma-1(ΔICD)/dma-1(ΔLRR)* and *dma-1(ΔICD)/dma-1(ΔLRR)* mutant animals at 1-day-old adult stage. Scale bar: 50 µm. **b**, Quantification of number of quaternary branches in a region 150 µm anterior to the PVD cell body. *P* = 0.0003 (wild-type versus wy908/wy1924), *P* < 0.0001 (*zac227; wy908* versus *zac227*), *P* < 0.0001 (*zac227; wy908* versus *zac227; wy908/wy1924*), *P* = 0.227 (*zac227* versus *zac227; wy908/wy1924*), *P* < 0.0001 (*zac227; wy908/wy1924* versus *wy908/wy1924*). **c**, Quantification of percentage of extended secondaries. Extended secondaries were measured as the percentage of secondary branches per worm that extended at least halfway to the tertiary dendrite line. *P* = 0.9497 (wild-type versus *wy908/wy1924*), *P* < 0.0001 (*zac227; wy90*8 versus *zac227*), *P* < 0.0001 (*zac227; wy908* versus *zac227; wy908/wy1924*), *P* = 0.9566 (*zac227* versus *zac227; wy908/wy1924*), *P* = 0.0007 (*zac227; wy908/wy1924* versus *wy908/wy1924*). **d**, Quantification of the total length of PVD dendrite, 150 µm anterior to the PVD cell body. *P* = 0.0134 (wild-type versus *wy908/wy1924*), *P* < 0.0001 (*zac227; wy908* versus *zac227*), *P* < 0.0001 (*zac227; wy908* versus *zac227; wy908/wy1924*), *P* = 0.0143 (*zac227* versus *zac227; wy908/wy1924*), *P* < 0.0001 (*zac227; wy908/wy1924* versus *wy908/wy1924*). For **b**–**d**, data are presented as mean ± s.e.m. *n* values within each bar. Statistical comparison was performed using Brown–Forsythe one-way ANOVA with Dunnett’s multiple comparisons test. *P < 0.05, ***P < 0.001, ****P < 0.0001. Schematic in **a** created in BioRender; Ho, X. Y. https://biorender.com/kx803o4 (2026).

Because DMA-1(*ΔLRR*) can support dynamic dendritic growth, we reasoned that its growth-promoting activity resides in the intracellular domain of DMA-1. DMA-1 and its co-receptor HPO-30 form a complex and use their cytosolic domains to directly recruit actin regulators such as TIAM-1 and the WAVE regulatory complex^15^. HPO-30 is a claudin-like tetraspanin that is highly enriched in PVD and is necessary for PVD dendrite outgrowth^13,14^. To understand how the cytosolic domains of DMA-1 and HPO-30 promote dendrite growth and branching, we generated a truncated endogenous DMA-1 lacking its intracellular domain (*dma-1(*ΔICD*)*) and an allele of HPO-30 lacking its C-terminal cytosolic domain (*hpo-30(*ΔICD*)*). When the cytosolic tails of both DMA-1 and HPO-30 were deleted (*hpo-30(*ΔICD*); dma-1(*ΔICD*)*), few 4° branches were formed, and the overall dendritic branches were dramatically reduced (Fig. 3a–d), indicating that the cytosolic domains of DMA-1 and HPO-30 are required to promote dendritic growth. Deletion of the HPO-30 (*hpo-30(*ΔICD*)*) alone results in a moderate increase in 4° branches and overall dendritic branches, confirming the redundancy between the two cytoplasmic domains (Fig. 3a–d).

The prevailing ligand-receptor model predicts that ligand binding to DMA-1 activates the actin organization through its cytosolic domain. We engineered DMA-1 as separate halves by creating one copy of extracellular-only *(ΔICD)*, and one copy of intracellular-only *(ΔLRR)* DMA-1, to disable the communication between the ecto- and intracellular domains. We analyzed *trans*-heterozygous *hpo-30(ΔICD); dma-1(ΔICD)/dma-1(ΔLRR)* animals generated by crossing *hpo-30(ΔICD); dma-1(ΔICD)* animals with *hpo-30(ΔICD); dma-1(ΔLRR)* animals. In these animals, HPO-30 lacks its cytosolic tail and DMA-1 exists as two separate proteins, one containing the extracellular domain and transmembrane domain (DMA-1(ΔICD)) and the other containing the transmembrane domain and cytoplasmic domain (DMA-1(ΔLRR)). Remarkably, we observed no significant difference in the number of 4° branches and the length of 2° branches in these animals compared with *hpo-30(ΔICD)* animals (Fig. 3a–c). The total dendrite length of the *hpo-30(ΔICD); dma-1(ΔICD)/dma-1(ΔLRR)* animals is slightly shorter than that of the *hpo-30(ΔICD)* animals, but longer than the *hpo-30(ΔICD); dma-1(ΔICD)* animals (Fig. 3d). This might be due to differences in the levels of DMA-1 expressed. More importantly, both *hpo-30(ΔICD); dma-1(ΔICD)/dma-1(ΔLRR)* and *dma-1(ΔICD)/dma-1(ΔLRR)* animals show ordered dendritic menorahs (Fig. 3a). These results argue that the functions of extracellular and intracellular domains of DMA-1 are distinct and can be achieved as separate proteins.

As cell surface receptors are presumed to first receive extracellular cues and subsequently activate intracellular signals, these results are surprising and support two notions. First, the cytoplasmic domain of DMA-1 promotes dendrite growth and branching in the absence of its extracellular domain. Second, the function of the DMA-1 receptor can be largely reconstituted as separate halves of DMA-1, each with distinct DMA-1 functions. The extracellular domain is required for the formation of the proper shape and morphology of dendrites while the intracellular domain promotes dendritic growth. From these results, we propose that ligand binding through the extracellular domain of receptor DMA-1 is not required for the intracellular domain to activate downstream signaling pathways to drive cytoskeletal rearrangement. Instead, ligand binding stabilizes dendrites and inhibits continuous dynamicity.

### Diffusible ligand-free DMA-1 is responsible for promoting dendritic growth

**Extended Data Fig. 2:**
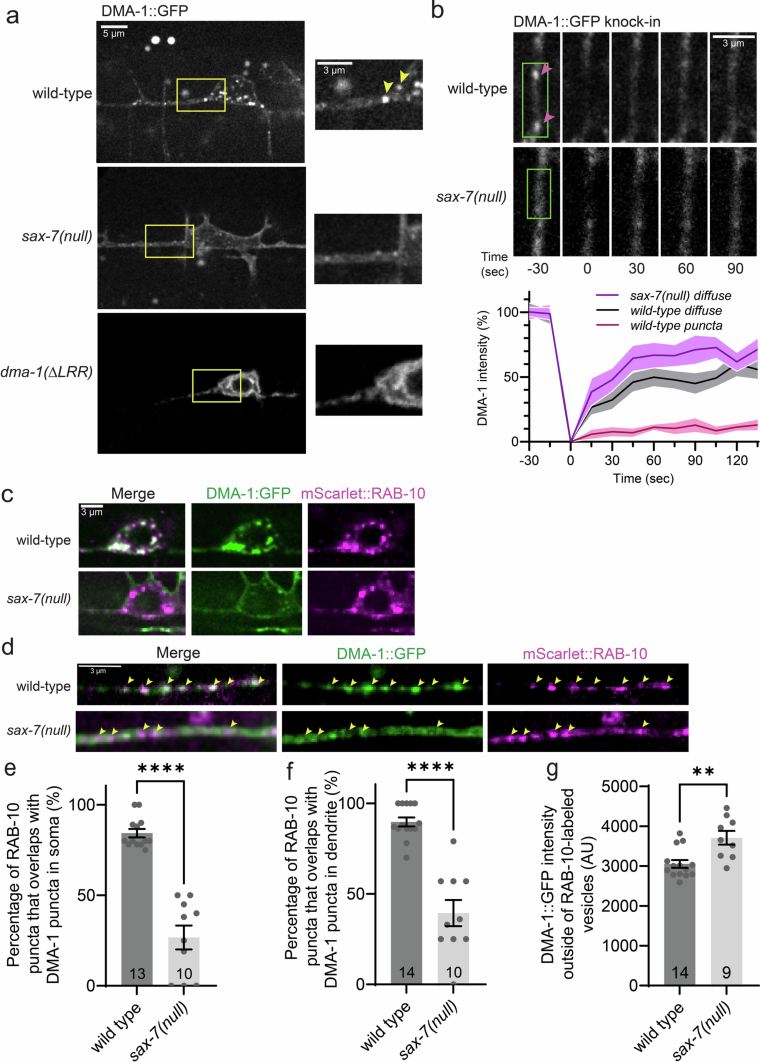
SAX-7 is required for the endosomal localization of DMA-1. **a**, Endogenous DMA-1::GFP knock-in in wild-type and *sax-7(null)* mutants and endogenous DMA-1(ΔLRR)::GFP knock-in mutants (n = 15 animals for all genotypes). Yellow boxes highlight the locations of magnified views on the right. Arrowheads indicate punctate structures representing putative endosomes. **b**, Fluorescence recovery after photobleaching in wild-type and *sax-7(null)* mutants expressing endogenous DMA-1::GFP. The green rectangle indicates the region of primary dendrite that was bleached at time = 0 sec. Arrowheads indicate DMA-1::GFP puncta. Data are presented as mean values +/− SD from independent animals (n = 36 diffuse regions analyzed from 14 individual wild-type worms; n = 20 puncta analyzed from 9 individual wild-type worms; n = 28 diffuse regions from 15 *sax-7(null)* individual animals). **c, d**, Colabeling of endogenous DMA-1::GFP with endogenous mScarlet:RAB-10 (mScarlet::RAB-10; expressed cell-specifically in PVD) in the soma c, and primary dendrite d, in the *sax-7(null)*. Arrowheads indicate locations of mScarlet::RAB-10 puncta. **e, f**, Quantification of the percentage of mScarlet::RAB-10 puncta that overlaps with DMA-1:GFP puncta in the soma e (*P* < 0.0001), and primary dendrite f (*P* < 0.0001), in the *sax-7(null)*. **g**, Quantification of DMA-1::GFP fluorescence intensity measured outside of mScarlet:RAB-10 puncta in the primary dendrite in *sax-7(null)*, P = 0.0027. For **e-g**, data are presented as mean values +/− SEM. n values within each bar. Statistical comparison was performed using a Mann-Whitney nonparametric t-test in **e-g**. **P < 0.01, ****P < 0.0001.

The observation that extracellular and intracellular functions of DMA-1 can be reconstituted as separate proteins strongly suggests that there are two populations of DMA-1 in vivo. Using endogenous DMA-1::GFP, we previously reported two pools of DMA-1 with distinct dynamics: a relatively mobile and diffuse pool outlining the PVD cell body and along the dendrites, and a punctate, internal vesicular pool both in soma and along the dendrites^20^ (Extended Data Fig. 2a). Consistent with previous findings, fluorescence recovery after photobleaching (FRAP) experiments showed that the diffuse signal recovered to about 50% of the pre-bleaching level within 45 s (Extended Data Fig. 2b). In contrast, the puncta show little recovery after photobleaching, consistent with DMA-1 being in internal vesicles with discontinuous membranes (Extended Data Fig. 2b).

To understand the identity of these DMA-1-containing vesicles, we examined RAB-10, a small GTPase localized to recycling endosomes which is essential for DMA-1 trafficking and PVD dendrite arborization^21,22^. We used a flippase-mediated labeling strategy^23^ to generate cell-specific, endogenously tagged mScarlet::RAB-10 in PVD and found that RAB-10 localizes to puncta distributed throughout the soma and dendrites (Extended Data Fig. 2c,d). In wild-type worms, we observed a high degree of colocalization between DMA-1 puncta and RAB-10-labeled vesicles in both the soma (Extended Data Fig. 2c,e) and dendrites (Extended Data Fig. 2d,f). Thus, the DMA-1 puncta likely represent a pool of DMA-1 on RAB-10-positive recycling endosomes. Many membrane receptors undergo ligand-induced endocytosis, which leads to desensitization of the ligand response^24^. Indeed, *sax-7(null)* mutants show fewer DMA-1 puncta both in the soma and along dendrites, while the diffuse DMA-1 on the cell membrane remains intact (Extended Data Fig. 2a). This distribution pattern of DMA-1 is similar to the localization of DMA-1(ΔLRR)::GFP, which might explain why *dma-1(ΔLRR)* and *sax-7(null)* mutants show similar dendrite phenotypes (Extended Data Fig. 2a). Furthermore, when we performed FRAP experiments on the diffusible pool of DMA-1 in *sax-7(null)* mutants, the rate of recovery of DMA-1::GFP was faster than wild-type animals (Extended Data Fig. 1b). The fluorescence recovery was also more complete in *sax-7(null)* mutants. To further illustrate this, we expressed mScarlet::RAB-10 in *sax-7(null)* mutant animals and quantified the percentage of DMA-1::GFP that overlaps with mScarlet::RAB-10, as well as the intensity of DMA-1::GFP outside of RAB-10. We observed that the percentage of DMA-1::GFP that colocalizes with mScarlet::RAB-10 both in the soma (Extended Data Fig. 2c,e) and dendrites (Extended Data Fig. 2d,f) was drastically reduced in *sax-7(null)* mutant compared with wild-type animals. In addition, there is an increase in DMA-1::GFP intensity outside of RAB-10 (Extended Data Fig. 2g), indicating that there is a larger portion of diffusible DMA-1. Together, these data are consistent with a model in which SAX-7 binding induces DMA-1 internalization into the RAB-10 endosomes.

### KPC-1 cleaves HPO-30

**Fig. 4:**
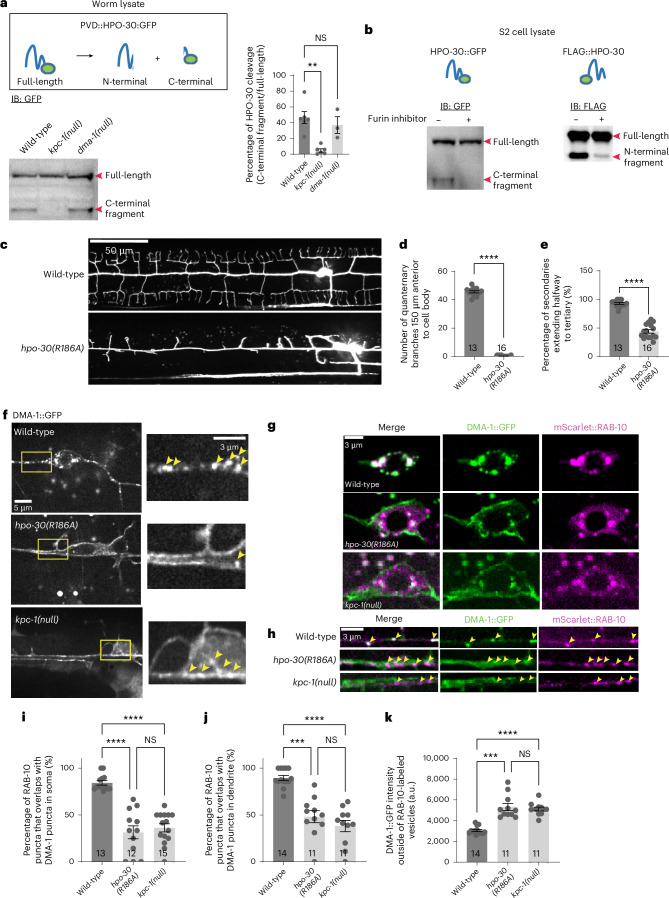
Cleavage of HPO-30 by KPC-1 facilitates DMA-1 recycling and is necessary for dendrite outgrowth. **a**, Western blot against GFP in worms expressing an HPO-30::GFP transgene expressed under a PVD promoter. Top: schematic of full-length HPO-30::GFP protein and putative N-terminal and C-terminal cleavage fragments. Bottom left: western blot using anti-GFP antibody on whole-worm lysate of wild-type, *kpc-1(null)* and *dma-1(null)* worms. Right: quantification of HPO-30 cleavage, calculated as intensity of C-terminal fragment out of total intensity. *P* = 0.0048 (wild-type versus *kpc-1(null)*) and *P* = 0.8446 (wild-type versus *dma-1(null)*). Data are presented as mean values ± s.e.m. Statistical comparison was performed using Brown–Forsythe one-way ANOVA with Dunnett’s multiple comparisons test (*n* = 6 blots for wild-type and *kpc-1(null)*; *n* = 3 blots for *dma-1(null)*). **b**, Western blot against GFP in cell lysates from *Drosophila* S2 cells expressing HPO-30::GFP (left) or FLAG::HPO-30 (right) constructs. Cells were grown in the absence (−) or presence (+) of the furin inhibitor decanoyl-RVKR-CMK. **c**, Representative images of PVD morphology in wild-type and *hpo-30(R186A)* mutants at the L4 stage. *hpo-30(R186A)* mutants were generated by CRISPR into the endogenous *hpo-30* locus. **d**, Quantification of number of quaternary branches in a region 150 µm anterior to the PVD cell body for *hpo-30(R186A)*. *P* < 0.0001 (wild-type versus *hpo-30(R186A)*). **e**, Quantification of percentage of extended secondaries in *hpo-30(R186A)*. *P* < 0.0001 (wild-type versus *hpo-30(R186A)*). In **d** and **e**, data are presented as mean ± s.e.m. *n* values within each bar. Statistical comparisons were performed using Welsh’s unpaired two-way *t*-test. **f**, Endogenous DMA-1::GFP in wild-type, *hpo-30(R186A)* and *kpc-1(null)*. Yellow rectangles indicate locations of magnified views on the right. Arrowheads indicate punctate structures representing putative endosomes. **g**,**h**, Colabeling of endogenous DMA-1::GFP with endogenous mScarlet::RAB-10 (mScarlet::RAB-10; expressed cell-specifically in PVD) in the cell bodies (**g**) and primary dendrite (**h**) of wild-type, *hpo-30(R186A)* and *kpc-1(null)* animals. Arrowheads indicate mScarlet::RAB-10 puncta. **i**,**j**, Quantification of the percentage of mScarlet::RAB-10 puncta that overlaps with DMA-1::GFP puncta in the cell bodies (**i**) (*P* < 0.0001 (wild-type versus *hpo-30(R186A)*), *P* < 0.0001 (wild-type versus *kpc-1(null)*) and *P* = 0.9125 (*hpo-30(R186A)* versus *kpc-1(null)*)) and primary dendrites (**j**) (*P* = 0.0001 (wild-type versus *hpo-30(R186A)*), *P* < 0.0001 (wild-type versus *kpc-1(null)*) and *P* = 0.5971 (*hpo-30(R186A)* versus *kpc-1(null)*)). **k**, Quantification of DMA-1::GFP fluorescence intensity measured outside of mScarlet::RAB-10 puncta (*P* = 0.0001 (wild-type versus *hpo-30(R186A)*), *P* < 0.0001 (wild-type versus *kpc-1(null)*) and *P* = 0.9396 (*hpo-30(R186A)* versus *kpc-1(null)*)). For **i**–**k**, data are presented as mean ± s.e.m. *n* values within each bar. Statistical comparison was performed using Brown–Forsythe one-way ANOVA with Dunnett’s multiple comparisons test. ***P < 0.001, ****P < 0.0001. IB, immunoblot.

Because SAX-7 and ligand binding is not required for DMA-1-mediated dendrite growth, we reason that there must be other mechanisms to activate DMA-1 for dendrite outgrowth. Our genetic approach found genetic interactions between *hpo-30* and *kpc-1*, both of which are required for PVD dendrite growth (more information in the Supplementary Text). Since KPC-1 is a furin-like proprotein convertase, we asked whether KPC-1 cleaves HPO-30 in PVD neurons. To test this, we generated worms expressing HPO-30 fused with GFP on the C-terminus (HPO-30::GFP) under the control of a PVD promoter (*ser2prom3::hpo-30::GFP*). Using western blot analysis with a GFP antibody, we found that HPO-30::GFP exists in both full-length HPO-30::GFP as well as a smaller fragment corresponding to a C-terminal cleavage fragment from worm lysates (Fig. 4a). Moreover, the shorter C-terminal fragment was not observed in *kpc-1(null)* mutants (Fig. 4a), indicating that HPO-30 cleavage is dependent on KPC-1. As HPO-30 is the co-receptor for DMA-1, we tested whether the DMA-1 is necessary for the cleavage of HPO-30. We observed that HPO-30::GFP cleavage is not affected in *dma-1(null)* mutants, suggesting that HPO-30 cleavage does not require DMA-1 (Fig. 4a). To further verify that furin-like proteases can cleave HPO-30, we expressed either C-terminally tagged HPO-30::GFP or N-terminally tagged FLAG::HPO-30 in *Drosophila* S2 cells and treated the cells with a furin inhibitor (decanoyl-RVKR-CMK). We then performed western blots using anti-GFP to detect cleaved C-terminal HPO-30 fragments or anti-FLAG antibodies to detect cleaved N-terminal HPO-30 fragments and observed that cleaved HPO-30 was greatly reduced in both cases (Fig. 4b). This suggests that KPC-1 is likely to cleave HPO-30 in both *C. elegans* and *Drosophila* cells.

### HPO-30 cleavage is required for dendritic growth and DMA-1 internalization

**Extended Data Fig. 3:**
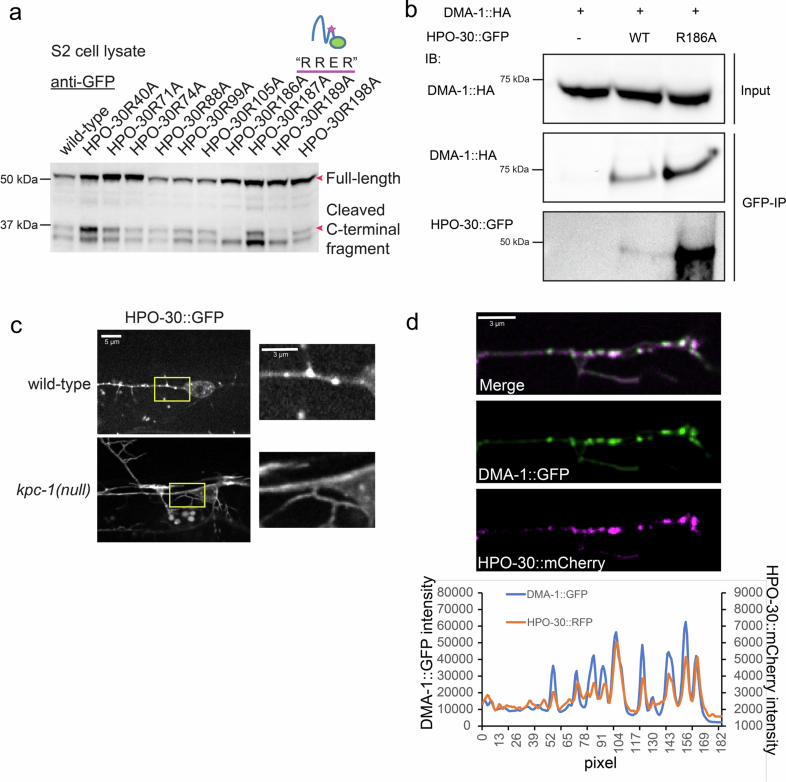
Uncleavable HPO-30(R184A) binds to DMA-1 and HPO-30 mislocalizes to the plasma membrane in *kpc-1(null)* mutants. **a**, Western blot against GFP in cell lysates from Drosophila S2 cells expressing wild-type HPO-30::GFP or HPO-30::GFP with individual arginine residues mutated to alanine as indicated. Top schematic represents the location of the R186A mutation in the second extracellular loop of HPO-30. **b**, Western blots showing co-IP between DMA-1::HA and HPO-30(WT)::GFP or HPO-30(R186A)::GFP expressed in S2 cells. Samples were immunoprecipitated with an anti-GFP nanobody. **c**, Endogenous HPO-30(WT)::GFP in wild-type and *kpc-1(null)*. Yellow rectangles indicate locations of magnified views on the right. **d**, Co-localization of DMA-1::GFP and HPO-30::mCherry in the PVD dendrites (top) and trace of either DMA-1::GFP intensity or HPO-30::mCherry intensity along the dendrite shows high co-localization between DMA-1 and HPO-30. n = 19 worms quantified.

To identify the cleavage site in HPO-30, we scanned the HPO-30 protein sequence for the furin recognition consensus motif ‘RXXR’^25^ and individually mutated these arginines to alanines (R to A). We systematically expressed these R-to-A constructs in S2 cells and assayed them for HPO-30::GFP cleavage. We found that either an R186A or R189A mutation eliminated the cleavage product (Extended Data Fig. 3a). Consistent with the canonical furin cleavage motif, these two residues are part of an ‘RRER’ sequence (residues 186–189) in the second extracellular loop of HPO-30 (Extended Data Fig. 3a), which is predicted to reside in the lumen of secretory space. The location of this cleavage site is also consistent with the size of the cleaved HPO-30 fragments (Fig. 4a).

Next, we generated animals containing R186A mutation in HPO-30 using CRISPR–Cas9. *hpo-30(R186A)* mutants showed a dramatic loss of dendrites similar to *hpo-30(null)*: quaternary branches were completely absent (Fig. 4c,d), while short secondary branches remained at about 50% of the level of wild-type controls (Fig. 4c,e and Extended Data Fig. 3b). These results indicate that HPO-30 cleavage is required for promoting dendritic growth.

As HPO-30 forms a complex with DMA-1 to promote dendritic growth, we sought to understand how HPO-30 cleavage activates this receptor complex. First, we performed co-immunoprecipitation experiments with DMA-1 tagged with HA along with either wild-type or R186A HPO-30-GFP in *Drosophila* S2 cells, and observed that uncleavable HPO-30(R186A) can still bind to DMA-1 (Extended Data Fig. 3b), suggesting that the HPO-30–DMA-1 complex is still intact and does not require HPO-30 cleavage.

We then asked whether HPO-30 cleavage affects DMA-1 localization. To do so, we examined DMA-1::GFP in *hpo-30(R186A)* and *kpc-1(null)* mutants where HPO-30 cleavage does not occur. Remarkably, punctate internal DMA-1::GFP signals were largely reduced in the soma and along the dendrite, but the plasma membrane signal was increased in both *hpo-30(R186A)* and *kpc-1(null)* animals (Fig. 4f). Further, the colocalization between DMA-1::GFP and mScarlet::RAB-10 in both *hpo-30(R186A)* and *kpc-1(null)* animals is reduced both in the soma (Fig. 4g,i) and along the dendrite (Fig. 4h,j). In addition, there was an increase in diffuse DMA-1 signal in both *hpo-30(R186A)* and *kpc-1(null)* animals (Fig. 4k). These data suggest that HPO-30 cleavage by KPC-1 promotes the internalization of DMA-1 into endosomes. Interestingly, when we visualized HPO-30::GFP in *kpc-1(null)* mutants, we also observed that HPO-30::GFP becomes more diffuse with fewer intracellular HPO-30 puncta compared with wild-type animals, similar to DMA-1::GFP (Extended Data Fig. 3c). These data are consistent with a model in which KPC-1 cleaves HPO-30 and induces the endocytosis of DMA-1–HPO-30 complex into recycling endosomes, which is essential for the growth-promoting functions of DMA-1 and HPO-30. Indeed, when we examined the localization of HPO-30 and DMA-1, they showed a high degree of colocalization in puncta along PVD dendrites (Extended Data Fig. 3d).

### Endosomal DMA-1 is important for PVD dendrite development

**Extended Data Fig. 4:**
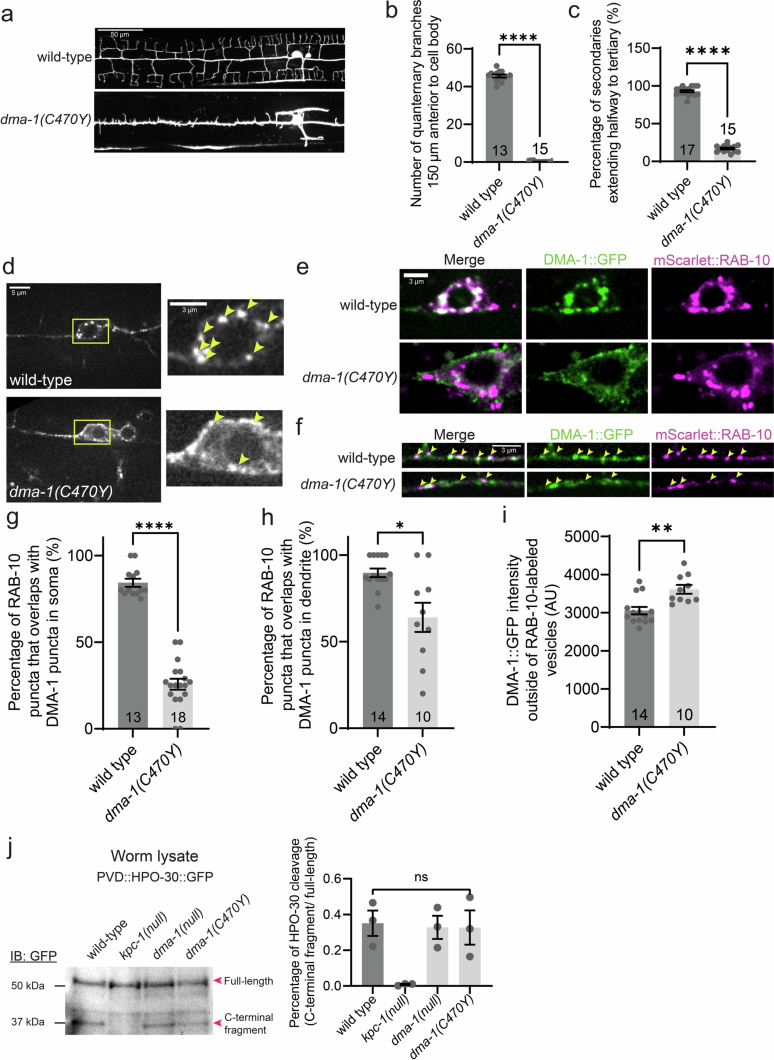
Endosomal DMA-1 is required for PVD dendrite morphogenesis. **a**, Representative images of PVD morphology in wild-type and *dma-1(C470Y)* mutants at the L4 stage. **b**, Quantification of number of quaternary branches in a region 150 µm anterior to the PVD cell body for *dma-1(C470Y)* animals (*P* < 0.0001). **c**, Quantification of percentage of extended secondaries in *dma-1(C470Y)* mutants (*P* < 0.0001). In **b,c, d**, Endogenous DMA-1::GFP knock-in in *dma-1(C470Y)* animals. Yellow rectangles indicate locations of magnified views on the right. Arrowheads indicate punctate structures representing putative endosomes. **e**, Colabeling of endogenous DMA-1::GFP with endogenous mScarlet::RAB-10 (mScarlet::RAB-10; expressed cell-specifically in PVD) in the cell bodies of *dma-1(C470Y)* mutant animals. **f**, Colabeling of endogenous DMA-1::GFP with endogenous mScarlet::RAB-10 (mScarlet::RAB-10; expressed cell-specifically in PVD primary dendrite of wild-type and *dma-1(C470Y)* animals. Yellow arrowheads indicate locations of mScarlet::RAB-10 puncta. **g, h**, Quantification of the percentage of mScarlet::RAB-10 puncta that overlap with DMA-1::GFP puncta in the cell bodies g (*P* < 0.0001), and primary dendrites h (*P* = 0.0142), in *dma-1(C470Y)* animals. **i**, Quantification of DMA-1::GFP fluorescence intensity measured outside of mScarlet::RAB-10 puncta in *dma-1(C470Y)* animals (*P* = 0.0015). For **b, c, g-i**, data are presented as mean values +/− SEM. n values within each bar. Statistical comparisons were performed using Welsh’s unpaired two-way *t* test. **j**, Western blot against GFP in worms expressing an HPO-30::GFP transgene expressed under a PVD promoter. Left: Western blot using anti-GFP antibody on whole-worm lysate of wild-type, *kpc-1(null)*, *dma-1(null)* and *dma-1(C470Y)* worms. Right: Quantification of HPO-30 cleavage, calculated as intensity of C-terminal fragment out of total intensity (P > 0.9999). Statistical comparison was performed using Brown-Forsythe one-way ANOVA with Dunnett’s multiple comparisons test (n = 3 blots). *P < 0.05, **P < 0.01, ****P < 0.0001.

Through a separate genetic screen, we identified a new allele of *dma-1*, *zac98* (*dma-1(C470Y)*), that contains a single C470Y point mutation in the extracellular domain of DMA-1. *dma-1(C470Y)* mutant animals displayed dramatic dendrite defects that are indistinguishable from the *kpc-1(null)* mutants but surprisingly distinct from *dma-1(null)* (Extended Data Fig. 4a–c), suggesting that the C470Y mutation does not simply cause a complete loss of *dma-1* function. When we assessed the localization of DMA-1(C470Y)::GFP in *dma-1(C470Y)* mutant animals, endogenous DMA-1(C470Y)::GFP exhibited increased plasma membrane localization and strongly reduced levels of internal puncta in the soma and the dendrites compared with the wild-type (Extended Data Fig. 4d), similar to *kpc-1(null)* and *hpo-30(R186A)* mutants (Fig. 4g–k).

Furthermore, DMA-1(C470Y)::GFP showed reduced colocalization with mScarlet::RAB-10 (Extended Data Fig. 4e–h) and increased diffuse signal along dendrites (Extended Data Fig. 4i). Additionally, we measured HPO-30::GFP cleavage in *dma-1(C470Y)* mutant animals and found that DMA-1 is cleaved in the same manner in wild-type animals. This suggests that DMA-1(C470Y)::GFP cannot be effectively internalized even though HPO-30 is cleaved by KPC-1 (Extended Data Fig. 4j). The C470Y mutation is located at a beta strand within the extracellular side of the juxtamembrane region of DMA-1, which is distant from the predicted ligand-binding region^19^. The location of the mutation is consistent with the idea that it does not eliminate ligand binding but instead impedes its internalization. These data further support the notion that DMA-1 needs to be internalized into endosomes for the proper formation of the PVD neuron.

**Extended Data Fig. 5:**
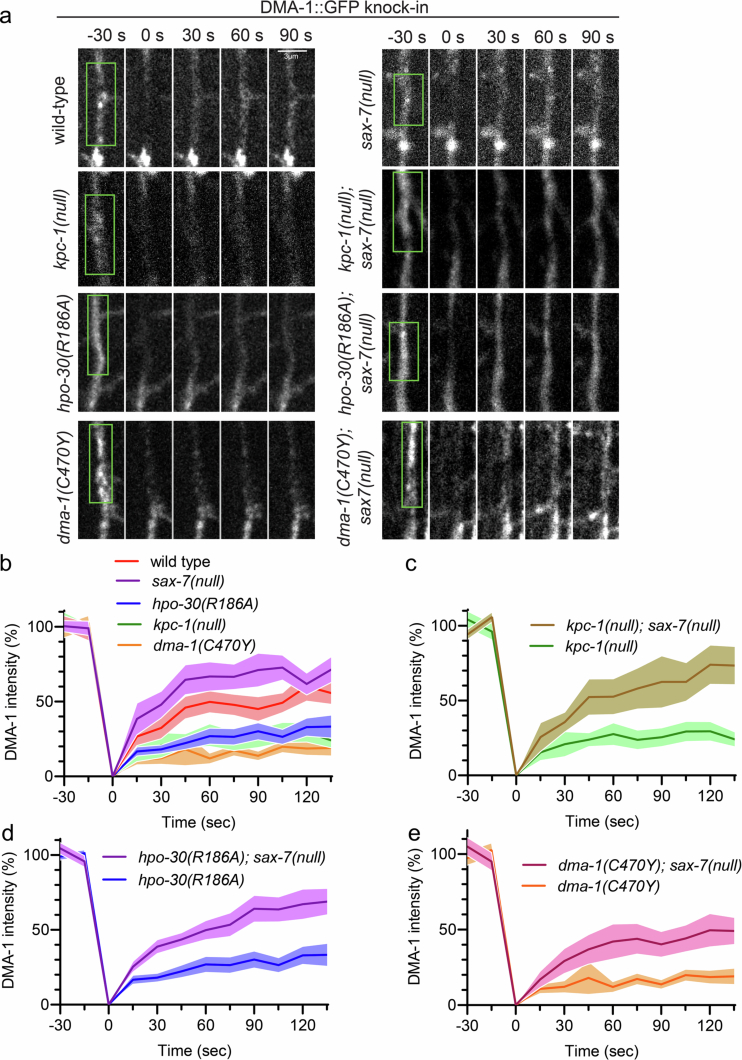
Endocytosis/recycling of DMA-1 provides a diffusible pool of DMA-1. **a**, Representative images of fluorescence recovery after photobleaching in the indicated genotypes expressing endogenous DMA-1::GFP. Green rectangle indicates the region of primary dendrite that was bleached at time = 0 s. Scale bar = 3µm. **b-e**, Fluorescence recovery after photobleaching analysis of endogenous DMA-1::GFP in the indicated genotypes showing in a. For **b-e**, data are presented as mean values +/− SD from independent animals. Wild-type: 36 diffuse regions from 14 animals, *kpc-1(null)*: 26 diffuse regions from 12 animals, *kpc-1(null); sax-7(null)*: 20 diffuse regions from 10 animals, *hpo-30(R186A)*: 23 diffuse regions from 12 animals, *hpo-30(R186A); sax-7(null)*: 28 diffuse regions from 15 animals, *dma-1(C470Y)*: 20 diffuse regions from 10 animals, *dma-1(C470Y); sax-7(null):* 28 diffuse regions from 14 animals.

**Extended Data Fig. 6:**
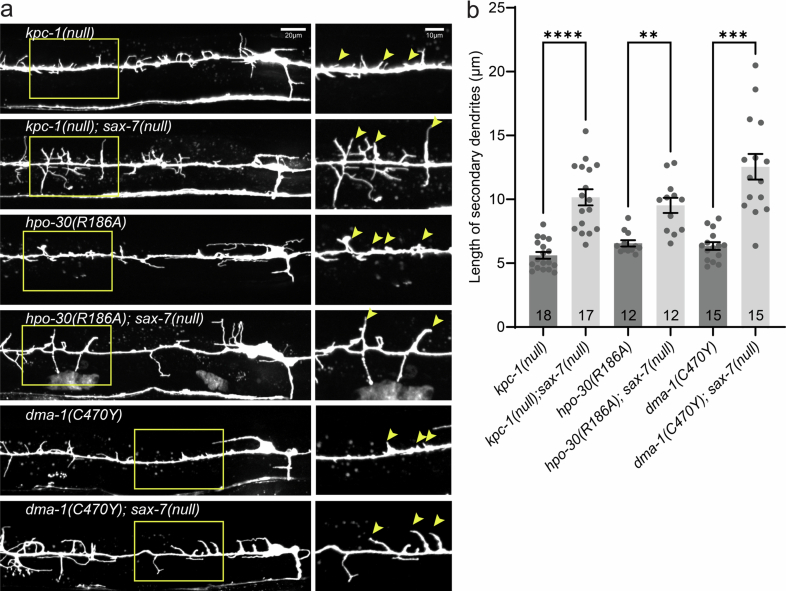
Removal of SAX-7 in *kpc-1(null), hpo-30(R186A)*, or *dma-1(C470Y)* animals increase dendritic growth. **a**, Representative images of PVD dendrite morphology at the L4 stage. **b**, Quantification of the mean length of all secondary dendrites in individual worms. *P* < 0.0001 (*kpc-1(null)* versus *kpc-1(null)*; *sax-7(null)*, *P* = 0.0044 (*hpo-30(R186A)* versus *hpo-30(R186A)*; *sax-7(null)*) and *P* = 0.0002 (*dma-1(C470Y)* versus *dma-1(C470Y)*; *sax-7(null)*). Data are presented as mean values +/− SEM. n values within each bar. Statistical comparison was performed using Brown-Forsythe one-way ANOVA with Dunnett’s multiple comparisons test. **P < 0.01, ***P < 0.001, ****P < 0.0001.

To further dissect the function of DMA-1 internalization in the formation of the PVD, we wondered whether the endocytic process is required to dissociate ligand-bound DMA-1 from its transmembrane ligand SAX-7 while it moves into endosomes to then be recycled onto the membrane as ligand-free DMA-1 to promote growth activity. To test this hypothesis, we performed FRAP analyses to measure the proportion of mobile DMA-1::GFP in mutants (*kpc-1(null)*, *hpo-30(R186A)* and *dma-1(C470Y)*) with defects in DMA-1 endocytosis, resulting in largely mislocalized DMA-1 on the plasma membrane. If DMA-1 endocytosis is required to form ligand-free DMA-1 on the plasma membrane, mutants with defects in DMA-1 endocytosis would have a reduction of mobile DMA-1 since ligand-free DMA-1 is more mobile than ligand-bound DMA-1 (Extended Data Fig. 2b). Indeed, we saw that DMA-1::GFP recovered poorly in these mutants after photobleaching, suggestive of a reduced mobile pool (Extended Data Fig. 5a,b). Because these mutants showed increased DMA-1 plasma membrane localization where DMA-1 interacts with its ligands, we wondered whether DMA-1 was immobilized through ligand binding. To test this, we removed the ligand SAX-7 in *kpc-1(null)*, *hpo-30(R186A)* and *dma-1(C470Y)* mutants by creating double mutants between *sax-7(null)* and each of the mutants. We then measured the proportion of mobile DMA-1 using FRAP. Strikingly, DMA-1::GFP recovers significantly faster and more completely in all three double mutants (*kpc-1(null); sax-7(null)*, *hpo-30(R186A); sax-7(null)* and *dma-1(C470Y); sax-7(null))* compared with the single mutants. This observation strongly indicates that ligand binding immobilizes DMA-1 in these three mutant backgrounds, resulting in PVD defects (Extended Data Fig. 5a,c–e). Moreover, the three double mutants which have an increased proportion of diffused DMA-1 exhibit longer dendritic branches than the corresponding single mutants (Extended Data Fig. 6a,b), suggesting that the binding between SAX-7 and DMA-1 immobilizes the receptor and inhibits branch growth. Together, these data are consistent with the two notions. First, the ligand-free DMA-1 present on the surface is generated by endosomal recycling and is required to promote dendritic growth. Second, while ligand binding inhibits growth and prevents retraction to achieve selective stabilization, it is also required for DMA-1 recycling to generate mobile ligand-free DMA-1 on the cell membrane necessary for dendritic growth.

### DMA-1 recycling promotes DMA-1 diffusion into nascent dendrites

**Fig. 5:**
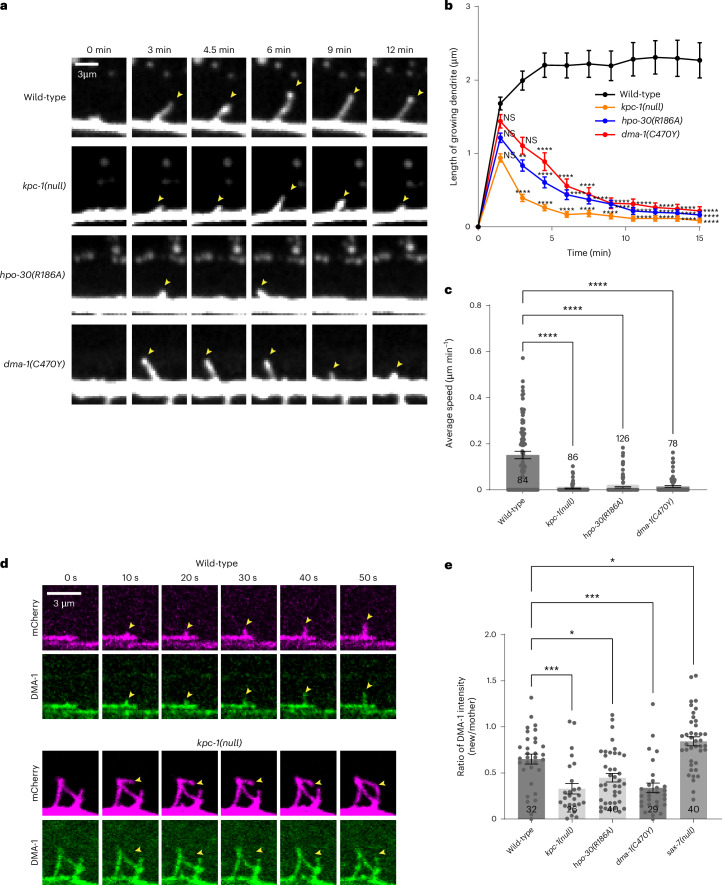
Endosomal DMA-1 is required for rapid recruitment of DMA-1 to newly formed dendrites. **a**, Time-lapse imaging of developing PVD secondary dendrites in wild-type, *kpc-1(null)*, hpo*-30(R186A)* and *dma-1(C470Y)* animals. Yellow arrowheads indicate growth and retraction events of the dendritic tips. **b**, A plot of growing secondary dendrite length over time. *P* ≥ 0.9999, <0.0001, <0.0001, <0.0001, <0.0001, <0.0001, <0.0001, <0.0001, <0.0001, <0.0001, <0.0001 (wild-type versus *kpc-1(null)*). *P* ≥ 0.9999, 0.0032, <0.0001, <0.0001, <0.0001, <0.0001, <0.0001, <0.0001, <0.0001, <0.0001, <0.0001 (wild-type versus *hpo-30(R186A)*). *P* ≥ 0.9999, 0.3783, <0.0001, <0.0001, <0.0001, <0.0001, <0.0001, <0.0001, <0.0001, <0.0001, <0.0001 (wild-type versus *dma-1(C470Y)*). Number of growing secondary dendrites used: wild-type = 84, *kpc-1(null)* = 86, *hpo-30(R186A)* = 126 and *dma-1(C470Y)* = 78. Data are presented as mean values ± s.e.m. Statistical comparison was performed using two-way ANOVA with Tukey’s multiple comparisons test. **c**, Quantification of the average speed of secondary dendrite growth in wild-type, *kpc-1(null)*, hpo*-30(R186A)* and *dma-1(C470Y)* animals. *n* values within each bar and more than four independent animals were imaged for each genotype. *P* < 0.0001 (wild-type versus *kpc-1(null)*), *P* < 0.0001 (wild-type versus *hpo-30(R186A)*), *P* < 0.0001 (wild-type versus *dma-1(C470Y)*). **d**, Time-lapse imaging of developing PVD dendrites with DMA-1::GFP (green) and mCherry (magenta) in wild-type and *kpc-1(null)* animals. **e**, Quantification of the ratio of DMA-1::GFP intensity (newly formed dendrite/mother dendrite) in wild-type, *kpc-1(null)*, hpo*-30(R186A)* and *dma-1(C470Y)* animals. *P* = 0.0006 (wild-type versus *kpc-1(null)*), *P* = 0.0225 (wild-type versus *hpo-30(R186A)*), *P* = 0.0004 (wild-type versus *dma-1(C470Y)*), *P* = 0.0401 (wild-type versus *sax-7(null*)). More than 13 independent animals were imaged for each genotype. For **c** and **e**, data are presented as mean ± s.e.m. *n* values within each bar. Statistical comparison was performed using Brown–Forsythe one-way ANOVA with Dunnett’s multiple comparisons test. *P < 0.05, **P < 0.01, ***P < 0.001, ****P < 0.0001.

Since DMA-1 functions as a guidance receptor, why does enhanced localization of DMA-1 on the plasma membrane and reduced endosomal localization lead to near-complete failure of dendrite morphogenesis? To further probe this question, we performed time-lapse imaging experiments to pinpoint the developmental deficits that led to the dramatic loss of dendrites in mutant animals with defects in DMA-1 recycling (*kpc-1(null)*, *hpo-30(R186A)* and *dma-1(C470Y)*). In wild-type animals, dendritic filopodia often undergo rounds of growth and retraction, which ultimately results in net growth (Fig. 5a–c). In contrast, in *kpc-1(null)*, *hpo-30(R186A)* and *dma-1(C470Y)* mutants with depleted endosomal DMA-1, short filopodia initiated normally but failed to extend beyond 1.5 µm in length. These short filopodia retracted within a few minutes, resulting in an overall slower growth speed (Fig. 5a–c). This further suggests that the reduction in mobile ligand-free DMA-1 impedes dendritic growth.

**Fig. 6:**
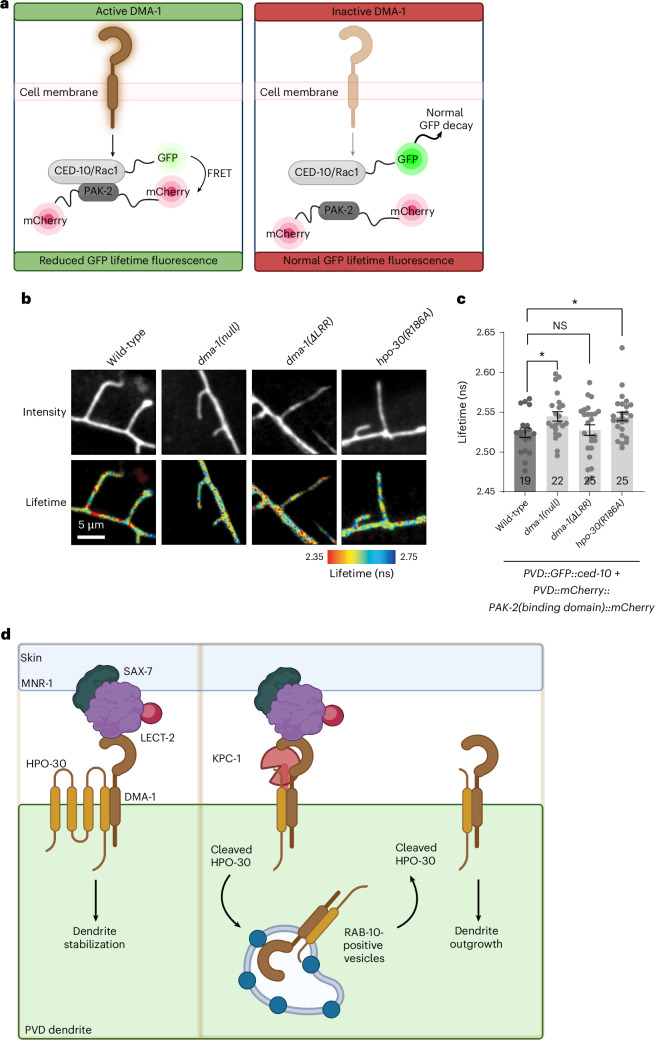
Ligand binding inhibits DMA-1 activation. **a**, Schematic of how interaction of CED-10::GFP and PAK-2::mCherry leads to fluorescence resonance energy transfer of GFP and mCherry leading to reduced GFP lifetime fluorescence. Reduced GFP lifetime fluorescence indicates downstream signaling while normal GFP decay indicates no CED-10 and PAK-2 interaction and hence no downstream signaling. **b**, Representative Δlifetime images of wild-type, *dma-1(null)*, *dma-1(∆LRR)* and *hpo-30(R186A)*. **c**, Quantification of GFP lifetime (ns) in wild-type, *dma-1(null)*, *dma-1(∆LRR)* and *hpo-30(R186A)* animals expressing GFP::CED-10 and mCherry::PAK-2(binding domain). *P* = 0.107 (control versus *dma-1(null)*), *P* = 0.9993 (control versus *dma-1(ΔLRR)*), *P* = 0.0847 (control versus *hpo-30(R186A)*). Data are presented as mean ± s.e.m. *n* values within each bar. Statistical comparison was performed using Brown–Forsythe one-way ANOVA with Dunnett’s multiple comparisons test. *P < 0.05. **d**, Stereotyped dendritic arbors require the recycling of ligand-free DMA-1 through HPO-30 cleavage and selective stabilization through ligand-bound DMA-1. Proper dendritic morphogenesis requires rounds of dendrite outgrowth and stabilization. Dendrite stabilization requires the binding of ligands to the HPO-30–DMA-1 complex. Dendritic outgrowth is dependent on endocytic recycling of DMA-1 to generate mobile ligand-free DMA-1. The activation of DMA-1 recycling requires ligand binding of DMA-1 to its ligand and cleavage of HPO-30 by KPC-1. Schematics in created in BioRender: **a**, Ho, X. Y. https://biorender.com/kx803o4 (2026); **d**, Ho, X. Y. https://biorender.com/kx803o4 (2026).

**Extended Data Fig. 7:**
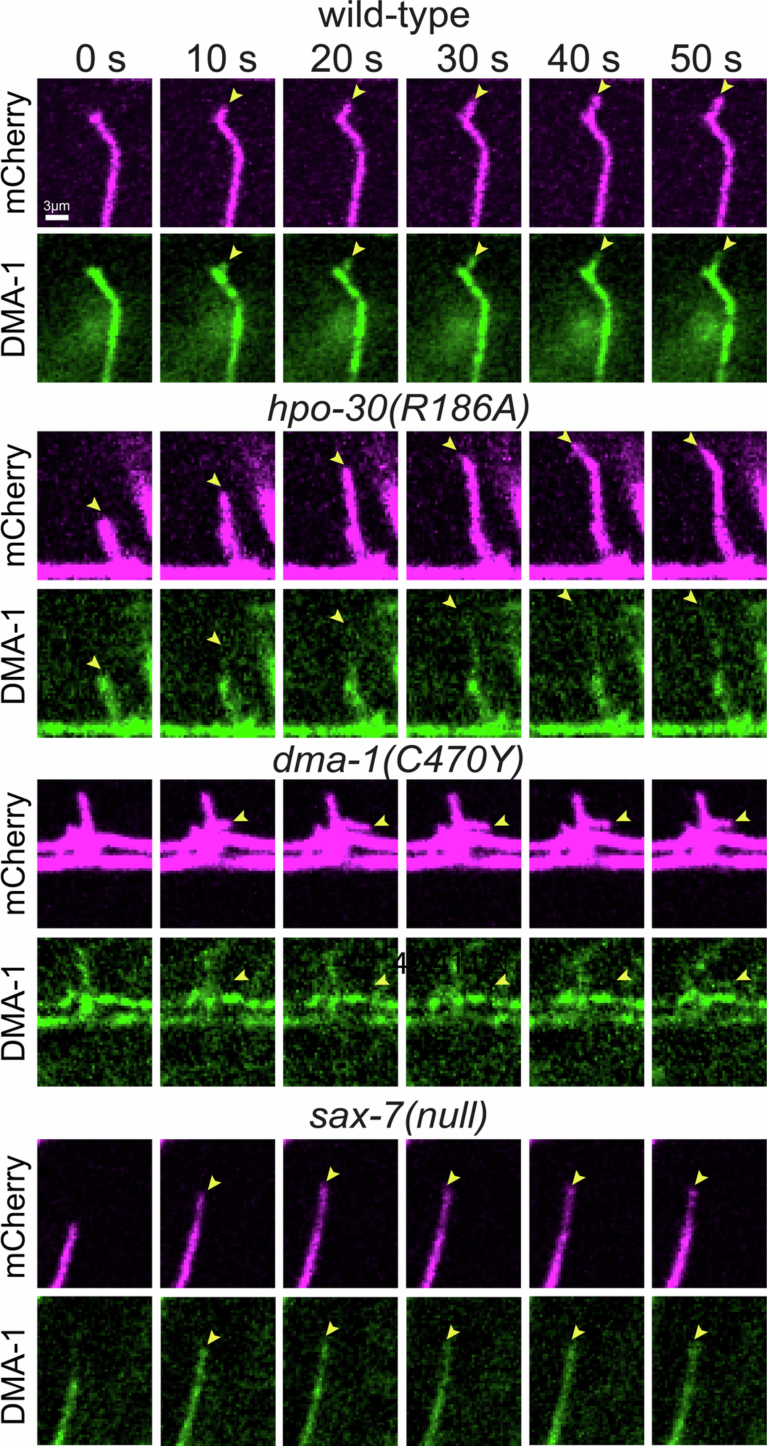
Delayed enrichment of DMA-1::GFP in the nascent filopodia of *hpo-30(R186A)* and *dma-1(C470Y*) mutants. Time-lapse imaging of developing PVD dendrites with DMA-1::GFP (green) and PVD marker (*ser2prom3::mCherry* (magenta)) in wild-type, *hpo-30(R186A)*, *dma-1(C470Y)* or *sax-7(null)* animals. Arrowheads point to the tip of outgrowing dendritic branches.

**Extended Data Fig. 8:**
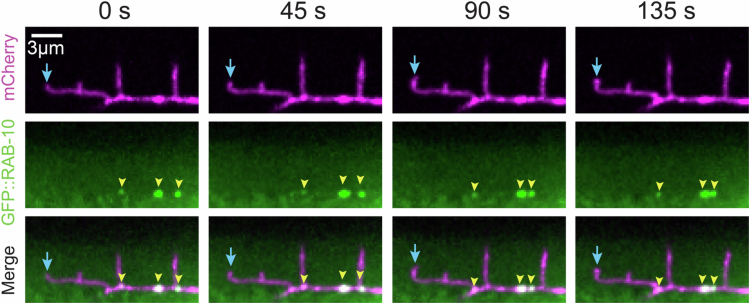
Endogenous GFP::RAB-10 is not localized in the growing PVD dendrites. Time-lapse imaging of developing wild-type PVD dendrites with GFP::RAB-10 (green) and (*ser2prom3::mCherry* (magenta)) in wild-type animals. Blue arrows indicate growing dendrites. Yellow arrowheads indicate GFP::RAB-10 puncta.

Next, we wondered whether this reduction in overall dendritic growth speed is due to the lack of DMA-1 in the newly generated filopodia. To characterize the presence of DMA-1 in nascent filopodia, we performed time-lapse imaging in animals expressing endogenous DMA-1::GFP and a PVD marker (*ser2prom3::mCherry*) and asked how quickly DMA-1::GFP enters a newly formed filopodia. This is quantified by calculating the ratio between the intensity in nascent filopodia and the intensity in the mother branch at 7.5 s after filopodia formation. In wild-type animals, DMA-1::GFP is enriched in the nascent filopodia within seconds after filopodia growth (Fig. 5d). However, in *kpc-1(null), hpo-30(R186A)* and *dma-1(C470Y)* mutants, it took significantly longer for DMA-1::GFP to appear and to be enriched in the nascent filopodia (Fig. 5d and Extended Data Fig. 7). *kpc-1(null)*, *hpo-30(R186A)* and *dma-1(C470Y)* mutants showed reduced enrichment of DMA-1 on nascent branches compared with wild-type animals (Fig. 5d,e and Extended Data Fig. 7). These data suggest that while the DMA-1 recycling mutants have higher levels of DMA-1 on their plasma membrane, DMA-1 is not rapidly recruited to nascent dendritic filopodia. Without DMA-1 in the nascent filopodia, the branches retract, leading to a lack of dendritic arbors. Our data suggest that ligand-free DMA-1 can laterally diffuse into nascent filopodia to facilitate dendrite growth (Fig. 5d,e). However, because of the striking colocalization between punctate DMA-1 and recycling endosomes, it is possible that exocytosis of DMA-1-containing recycling endosomes contributes to the enrichment of DMA-1 at the tips of the nascent filopodia. To test this idea, we used endogenously labeled GFP::RAB-10 to track the localization and dynamics of RAB-10-positive endosomes during filopodia formation. We found that RAB-10 puncta were distributed sparsely along the dendrite, with no obvious enrichment at the sites of filopodia formation (Extended Data Fig. 8). In most of the de novo filopodia formation events, there was no obvious RAB-10-positive puncta localized near or inside of the nascent filopodia, suggesting that nascent filopodia formation does not require local RAB-10 endosomal exocytosis. These genetic experiments support the idea that ligand-free receptor promotes branching while ligand-bound receptors inhibit dendrite growth. To address how ligand binding modulates receptor signaling, we established an in vivo signaling assay in *C. elegans* to measure the downstream events of DMA-1 activation. Previous genetic dissections showed that Rac activity is required for PVD dendrite morphogenesis^3,13^. To measure DMA-1 signaling, we co-expressed CED-10/RAC1::GFP together with mCherry::PAK-2(binding domain)::mCherry in PVD and measured fluorescence resonance energy transfer in PVD dendrites using fluorescence lifetime imaging microscopy (FLIM) (Fig. 6a). To test whether ligand binding is required for activating downstream signaling of DMA-1, we compared FLIM signals in developing dendrites from wild-type controls and *dma-1(null)*, *dma-1(∆LRR)* and *hpo-30(R186A-uncleavable)* mutants (Fig. 6b,c). We hypothesized that animals containing wild-type DMA-1 would have an intact growth/signaling function as well as cell adhesion function. *dma-1(null)* mutants would have neither function. We hypothesized that *dma-1(∆LRR)* mutants would retain the signaling function but not adhesion function. In *hpo-30(R186A-uncleavable)*, our model predicts that the signaling function is lost because DMA-1 is trapped on plasma membrane, cannot be recycled and is constitutively bound to ligands. Our results showed that *dma-1(null)* has longer fluorescence lifetime than wild-type control, suggestive of lower Rac1 activity (Fig. 6b,c). Interestingly, *dma-1(∆LRR)* mutants showed similar FLIM signals to wild-type animals, indicating that DMA-1 signaling is active in *dma-1(∆LRR)* animals. Furthermore, *hpo-30(R186A-uncleavable)* mutants showed longer lifetime and were indistinguishable from *dma-1(null)* (Fig. 6b,c). Together, these results support the notion that ligand binding inhibits growth and that the recycling of DMA-1 by HPO-30 is required for DMA-1 signaling.

Collectively, these data reveal a new model for how ligand-receptor systems mediate stochastic dendrite growth to build stereotyped dendritic arbors (Fig. 6d). First, SAX-7 interacts with DMA-1 on the plasma membrane to stabilize dendritic branches and build dendritic arbors based on SAX-7 localization. Second, SAX-7 also induces DMA-1 endocytosis and drives DMA-1 into the RAB-10 endosomes. Through this process, DMA-1 dissociates from the transmembrane SAX-7 and becomes ligand-free. Third, once the receptor is recycled on the plasma membrane, the ligand-free DMA-1 diffuses into the nascent filopodia and promotes dynamic, stochastic growth. The cycle repeats when ligand-free DMA-1 binds to SAX-7. This new model provides a molecular mechanism to integrate the stochastic growth during dendritic morphogenesis and the stereotyped dendritic arbor. Ligand-free receptors promote stochastic, dynamic growth, while ligand-bound receptors stabilize dendrites and inhibit dynamic growth to achieve the arbor shape.

## Discussion

During dendritic morphogenesis, intrinsic growth molecules and extrinsic guidance cues act in concert to determine the proper size and shape of a neuron. Our data suggest that DMA-1 functions as an adhesion molecule to stabilize growing dendrites through its extracellular ligand-binding domain, but also promotes dendritic growth through its cytoplasmic domain. We show that this growth-promoting function is dependent on KPC-1 cleavage of HPO-30 which activates the DMA-1–HPO-30 receptor complex. We demonstrate that a pool of diffusible DMA-1, generated by recycling endosomes, rapidly diffuse into nascent filopodia to promote dendritic growth.

Dynamic imaging of dendrite branching in *Drosophila* neurons has shown that dendrite tips exhibit stochastic growth, pause and retraction^6,7,16,26^, and computational modeling suggests that this dynamic nature and the rapid transitions between growth and retraction shape and maintain dendritic morphology^27^. In *C. elegans*, the developing PVD dendrites also exhibit continuous growth and retraction^7,16,28^. Our results elucidated the molecular forces behind growth and retraction: stochastic intrinsic growth mediated by ligand-free DMA-1 followed by selective growth inhibition and branch stabilization through ligand-bound DMA-1. This model challenges the conventional view in neuronal morphogenesis where ligand binding to cell surface receptors induces a conformational change in the cytoplasmic tail to drive axon and dendrite branching. This model predicts higher dendritic dynamicity in low-ligand areas and low dynamicity in high-ligand regions. Indeed, the distribution of SAX-7 is enriched in tertiary and quaternary branches which exhibit less stochastic growth and retraction^29,30^.

How are dendrite growth and stabilization coordinated by DMA-1? We propose that DMA-1 internalization and recycling connect the growth and stabilization. When DMA-1 is endocytosed, it dissociates from its bound transmembrane ligand, SAX-7/L1CAM. Once it is reinserted on the plasma membrane through recycling endosomes, DMA-1 is now ligand-free and can diffuse into nascent filopodia, where it is ready for binding to available ligands. We found three genetic manipulations (*kpc-1(null), hpo-30(R186A)* and *dma-1(C470Y)*) that reduce the endocytosis of DMA-1, causing DMA-1 to accumulate on the plasma membrane. In all three mutants, dendrite growth is severely blocked due to the absence of DMA-1 in the nascent filopodia. Furthermore, previous studies showed that the reinsertion of DMA-1 onto the membrane requires the small GTPase RAB-10 and the exocyst complex^22^. In *rab-10* mutants, DMA-1 and HPO-30 accumulate in intracellular vesicles and fail to be recycled onto the plasma membrane^21,22^, and *rab-10* mutants also exhibit severe PVD outgrowth defects^21,22^. When the endocytosis or recycling of DMA-1 is perturbed, DMA-1 becomes constitutively localized to the plasma membrane where it is immobilized through binding to its ligand (SAX-7/L1CAM). It is worth noting that DMA-1 colocalizes with RAB-7 instead of RAB-10 axon initial segment, suggesting that the RAB-10-mediated recycling of DMA-1 is a compartment-specific membrane trafficking behavior related to dendrite growth and branching^20^.

How is the endocytosis of DMA-1 regulated? Our data indicate that KPC-1 cleavage of HPO-30 activates the DMA-1–HPO-30 receptor complex to promote dendritic morphogenesis. While it has been reported that KPC-1 functions cell-autonomously in PVD^16,17^ and DMA-1 is not required for HPO-30 cleavage by KPC-1 (Fig. 4a) to promote dendrite formation, it is plausible that ligand interaction with DMA-1 triggers HPO-30 cleavage which then enables the endocytosis. Consistently, we found increased membrane DMA-1::GFP and reduced endosomal DMA-1::GFP in the noncleavable *hpo-30(R186A)* mutant allele (Fig. 4f,g). These data indicate that the full-length HPO-30 prevents internalization, but this effect is negated once KPC-1 cleaves HPO-30. We speculate that when DMA-1 binds to full-length HPO-30, its lateral diffusion and endocytosis are inhibited, possibly due to claudin’s ability to form *cis* interactions with itself^31^. When DMA-1 binds to cleaved HPO-30, it is then able to be endocytosed and recycled back to the plasma membrane, providing a ligand-free receptor that can diffuse into newly formed filopodia. HPO-30 has also been found to maintain surface levels of levamisole-dependent AChRs at neuromuscular junctions^31^, suggesting the regulation of surface receptor levels may be a generalizable function of HPO-30 and perhaps other claudins.

Similar to many sensory receptors on the cell surface, guidance receptors undergo endocytosis and recycling. Receptor endocytosis has been shown to dynamically regulate the level of guidance receptors on the plasma membrane and hence the sensitivity to ligands^8,30^. Receptor-containing endosomes can serve as signaling hubs, which has been extensively described for a variety of receptor classes, including neurotrophin TrkA receptors^32^. TrkA receptor on the signaling endosomes continues to be active and associates with downstream signaling components^32^. As another example, G-protein-coupled receptors can form signaling endosomes after binding to beta-arrestins^33–35^. In this study, we uncovered a previously unknown function of endocytosis to remove transcellular ligands of membrane receptors through endosomal recycling. Future studies will be necessary to test the generality of this model.

## Methods

### *C. elegans* strains and plasmids

*C. elegans* animals were grown on nematode growth medium plates using OP50 *Escherichia coli* as a food source and maintained according to standard procedure unless otherwise noted^36^. Before imaging, animals were raised at 20 ºC for at least one generation. N2 Bristol worms were used as the wild-type strain*. C. elegans* plasmids were generated in a pSMdelta vector backbone.

### Ethyl methane sulfonate mutagenesis and forward genetic screens

For the *kpc-1(xr58)* genetic modifier screen, *kpc-1(xr58); wyIs592* worms were mutagenized at the L4 stage with 50 mM ethyl methane sulfonate (EMS). F2 animals were screened under a fluorescent compound microscope for PVD morphology defects resembling *kpc-1* full loss-of-function mutants, including short and disorganized secondary dendrites. The *wy1008* allele was isolated from a screen of 20,000 haploid genomes and mapped to the *hpo-30* gene using standard single nucleotide polymorphism mapping^37^. The *wy1008* allele carries a missense C-to-T point mutation flanked by the sequences CAAAAGCT and TCTGGCCT, resulting in an E100K amino acid substitution.

To identify additional mutants defective in PVD dendrite morphogenesis, *wyIs594* (*ser2prom3::myr-gfp*) animals at the L4 stage were mutagenized with 50 mM EMS. Five F1 animals were transferred into individual plates seeded with OP50. About 100 F2 animals from each plate were examined under a fluorescent compound microscope for abnormal PVD dendrite arborization. The *zac98* allele was isolated from a screen which covered about 30,000 haploid genomes. *zac98* failed to complement the *dma-1* null allele *wy686*. The *zac98* allele carries a missense G-to-A point mutation flanked by the sequences AATGTCCGAT and CGCGACACCA, resulting in a C470Y amino acid substitution. The *zac227* allele contains a T-to-A (T1315A) mutation which affects the splicing of intron 5 of the *hpo-30* gene.

### CRISPR–Cas9 genome editing

Endogenous labeling of DMA-1 and RAB-10, as well as the generation of the *wy1220*, *wy1437* and *wy1924* alleles, was done by injection of CRISPR–Cas9 protein complexes into the gonad. Injection mixes consisted of 1.525 μM ALT-R Cas9 protein (IDT), 1.525 μM tracRNA (IDT), 1.525 μM CRISPR RNA (crRNA, IDT) and repair templates consisting of 0.5 μM single-stranded DNA (IDT) or 0.15–2 μM double-stranded PCR template. Single-stranded DNA repair templates were used for engineering the point mutations in the *wy1220* and *wy1437* alleles and were designed with a 30-base pair homology arm flanking the edited region. Double-stranded PCR repair templates were used for fluorophore insertions. All repair templates either mutated the protospacer adjacent motif site or introduced silent mutations within the crRNA target sequence.

The *dma-1(ΔLRR)*, *wy1924* allele was engineered using CRISPR–Cas9 to delete the LRR domain of DMA-1. In total, 1,233 base pairs were deleted from *dma-1* (CTGCCATCTTTGGAAGTATT…AACAAGTGTACGTAGATGGA).

The *wy1437* allele was engineered using CRISPR–Cas9 to mimic a missense G-to-A point mutation, resulting in a C470Y point mutation that was originally isolated by an EMS screen. The *wy1924* allele was engineered using CRISPR–Cas9 to delete the LRR domain of DMA-1.

The following crRNAs were used:

*wy1616*: TGAAGAGCATGTCATACGGT

*wy1220*: TTTTACATAAATGGGTCCAA and GTTTTATCGAGAAGAGAACG

*wy1437*: GAATGACCTTCCACAAGCGATGG

*wy1924*: AGATCTAATACTTCCAAAGA and GTTGAACAAGTGTACGTAGA

### Sample preparation for western blotting

For western blots performed on S2 cell lysates: S2 cells were transfected with Pactin::hpo-30::gfp, Pactin::FLAG::hpo-30 or plasmids with R-to-A point mutations in Pactin::hpo-30::gfp (Supplementary Table 2). For cells grown in the presence of a furin inhibitor (Fig. 4b), 50 μM Decanoyl-RVKR-CMK (Tocris cat. no. 3501) was added to the S2 media during transfection. At 3 d after transfection, cells were lysed in RIPA buffer (Thermo Fisher cat. no. 89900) with 1x Halt Protease Inhibitor Cocktail (Thermo Fisher cat. no. 87786) for 30 min on ice. Cell lysates were spun at 13,000 rpm for 10 min at 4 °C. Supernatants were collected for western blot analysis in NuPAGE LDS Sample Buffer (Life Technologies cat. no. NP0007) supplemented with dithiothreitol (GoldBio cat. no. DTT10), and proteins were detected with a mouse antibody to GFP (1:2,000, Roche cat. no. 11814460001), mouse antibody to FLAG (1:1,000, Sigma cat. no. F3165) or mouse antibody to actin (1:5,000, Abcam cat. no. ab8224).

For western blots performed on worm lysates: Worms were collected from one 10-cm nematode growth medium plate for each worm strain by washing with M9 buffer. Worms were spun at 2,000 rpm and the worm pellet was washed with M9 1–3 times to remove residual bacteria. Worm pellets were lysed with an equal volume of NuPAGE LDS Sample Buffer (Life Technologies cat. no. NP0007) supplemented with dithiothreitol (GoldBio cat. no. DTT10), boiled for 10 min, then spun at 13,000 rpm for 10 min at room temperature. Supernatants were collected for western blot analysis using a mouse antibody to GFP (1:2,000, Roche cat. no. 11814460001) or mouse antibody to actin (1:5,000, Abcam cat. no. ab8224).

### Confocal imaging of *C. elegans*

All images were acquired at room temperature in live *C. elegans*. For images of mature PVD dendrite morphology, L4 and 1-day-old stage worms were anesthetized using 10 mM levamisole in M9 buffer and mounted on 3% agarose pads. Worms were then imaged on a 3i spinning disk microscope with a CSU-W1 spinning disk (Yokogawa) using a C-Apochromat ×40/0.9 NA water immersion objective, or a spinning disk microscope with a CSU-X1 spinning disk (Yokogawa) using a ×40 oil immersion objective. Images were acquired as z-stacks (0.75 μm per step, 15–18 steps), and maximum-intensity projections were used for analysis and data presentation.

For images of endogenous DMA-1 or RAB-10 protein localization, worms were similarly anesthetized and mounted, then imaged on a spinning disk microscope with a CSU-X1 spinning disk (Yokogawa) using a ×100 oil immersion objective. Single z-slices were chosen for all analyses.

For live time-lapse imaging of the developing PVD dendrite arbor and endogenous DMA-1:GFP during PVD dendrite growth, L3 or L4 stage worms were mounted onto a glass-bottom imaging dish (MatTek)^38^. Worms were picked into a 5 mM droplet of levamisole and covered with a 5% agarose pad. Worms were then imaged on a spinning disk microscope with a CSU-X1 spinning disk (Yokogawa). For developing PVD dendrite arbors, images were acquired every 90 s as z-stacks (0.75 μm per step, 12 steps) using a ×40 oil objective and maximum-intensity z-projections were used for analysis and data presentation. For endogenous DMA-1::GFP during PVD dendrite outgrowth, images were captured using a ×100/1.4 NA objective, 488-nm and 561-nm lasers, with a time interval of 2.5 s for a duration of up to 8 min. FIJI plugin StackReg was used for the alignment of a stack of images to generate movies. All images were analyzed using FIJI (ImageJ version 1.54).

### FRAP of DMA-1::GFP

FRAP assays were performed on a 3i spinning disk microscope with a CSU-W1 spinning disk (Yokogawa) using a C-Apochromat ×63/1.2 NA water immersion objective with ×2 magnification enhancer. Photobleaching was performed using a Vector x,y scanner (3i) and single z-slice images were collected every 15 s.

FRAP analysis was performed in ImageJ/Fiji. Images were cropped and registered using the StackReg plugin and corrected for photobleaching resulting from time-lapse imaging. FRAP intensity within the photobleached region was normalized to the average intensity of two background measurements taken before bleaching (100%), and the intensity immediately after bleaching (0%).

### Quantification of dendrite growth dynamics and enrichment of DMA-1::GFP in nascent filopodia

To measure the speed of dendrite growth, all the 2° dendrites that initiated protrusion from 1° branches were selected for quantification. Each growing secondary dendrite was tracked for 15 min after initiation. The frame captured just before the detection of the dendrite protruding from the 1° branch was designated as time 0 min.

To measure the enrichment of DMA-1::GFP in nascent filopodia, all the filopodia protrusions captured in the movies were selected for quantification. We define the frame right before the protrusion occurs as 0 s. Lines were drawn along the nascent filopodia at time point 7.5 s and their mother dendrite at 0 s. Adjacent regions in the same frame were used as the background. Enrichment of DMA-1::GFP in nascent filopodia = (mean intensity of nascent filopodia − mean intensity of background)/(mean intensity of mother dendrite − mean intensity of background).

### Fluorescence lifetime imaging measurements

The two-photon microscope was built as previously described^39^ and the in vivo two-photon microscope was built based on the open-access design of the Modular In vivo Multiphoton Microscopy System (MIMMS) from Howard Hughes Medical Institute Janelia Research Campus (https://www.janelia.org/open-science/mimms). FLIM capacity was added as previously described^40,41^. Briefly, a photodiode (Thorlabs cat. no. FDS010) was added to detect the arrival of the laser pulses. The output of a fast photomultiplier tube (Hamamatsu cat. no. H7422PA-40 or cat. no. H10769PA-40) was compared with laser pulse timing using a TCSPC-730 (Becker and Hickl) time-correlated single photon counting board. Hardware and data acquisitions using the TCSPC-730 were controlled by the ScanImage software (Vidrio) integrated with a modified add-on called FLIMimage, which was written in MATLAB and was kindly provided by R. Yasuda. Fluorophores were excited with a pulsed 80 MHz Titanium–Sapphire laser at 960 nm. The fluorescence emissions for these were unmixed using a Chroma 565DCXR dichroic mirror and Semrock FF01-630/92 and Chroma HQ510/70 band pass filters. Comparisons were done with an unpaired *t*-test using a Bonferroni correction for multiple comparisons. L3 stage worms were mounted onto a glass-bottom imaging dish (MatTek). Worms were picked into a 5 mM droplet of levamisole and covered with a 5% agarose pad.

### Statistical analysis

Data are represented as mean ± s.e.m. with corresponding data points as indicated in each figure legend. Statistical analyses were performed using either two-sided Student’s Welsh’s *t*-test or one-way analysis of variance (ANOVA) for multiple comparisons. All statistical analyses and graph constructions were performed using Prism 10.5.10 software (GraphPad Software). For very small *P* values (*P* < 0.0001) GraphPad does not provide exact numbers, which are therefore reported as *P* < 0.0001 versus respective control conditions. No statistical methods were used to pre-determine sample sizes, but our sample sizes are similar to those reported in previous publications^3,11,16,19–21^. Statistical analysis was chosen based on data distribution and model assumptions. All animals were randomly selected, and no animals were excluded from the analyses. All data collection and analyses were not performed blind to the conditions of the experiments, except experiments from FLIM imaging.

#### Ethics statement

The only animals used in this study are *C. elegans* nematodes, which are not subject to animal welfare regulations. No ethical approval was required.

### Reporting summary

Further information on research design is available in the Nature Portfolio Reporting Summary linked to this article.

## Online content

Any methods, additional references, Nature Portfolio reporting summaries, source data, extended data, supplementary information, acknowledgements, peer review information; details of author contributions and competing interests; and statements of data and code availability are available at 10.1038/s41593-026-02278-0.

## Supplementary information

Supplementary Information Supplementary Text, Fig. 1 and figure legend, and Tables 1 and 2.

Reporting Summary

Supplementary Video 1 Growing PVD dendrites at L3 stage.

Supplementary Video 2 Growing PVD dendrites at L4 stage.

Supplementary Video 3 Growing PVD dendrites of dma-1 mutants at L3 stage.

Supplementary Video 4 Growing PVD dendrites of sax-7 mutants at L3 stage.

Supplementary Video 5 Growing PVD dendrites of dma-1(LRR) mutants at L3 stage.

Supplementary Video 6 Growing PVD dendrites of lect-2 mutants at L3 stage.

Supplementary Video 7 Growing PVD dendrites of dma-1 mutants at L4 stage.

Supplementary Video 8 Growing PVD dendrites of sax-7 mutants at L4 stage.

Supplementary Video 9 Growing PVD dendrites of dma-1(LRR) mutants at L4 stage.

Supplementary Video 10 Growing PVD dendrites of lect-2 mutants at L4 stage.

Supplementary Video 11 Growing PVD dendrites towards sax-7 strips.

## Source data

Source Data Unprocessed western blots.

## Data Availability

All relevant data supporting the key findings of this study are available within the article and its Supplementary Information or from the corresponding author upon reasonable request. Source data are provided with this paper.

## Code availability

No custom software codes were used in the data analysis for this paper.
