# Supplementary material for: Stochastic growth and ligand–receptor interaction-mediated stabilization generate stereotyped dendritic arbors
Source: Nat Neurosci. 2026 May 4;29(6):1313–26. doi: 10.1038/s41593-026-02278-0 (PMC13246438; doi:10.1038/s41593-026-02278-0)
Supplement: Supplementary file 1 — Supplementary Text, Fig. 1 and figure legend, and Tables 1 and 2. [file 41593_2026_2278_MOESM1_ESM.pdf]

nature neuroscience

Supplementary information

https://doi.org/10.1038/s41593-026-02278-0

# Stochastic growth and ligand–receptor interaction-mediated stabilization generate stereotyped dendritic arbors

In the format provided by the authors and unedited

Supplemental Text

**KPC-1 cleaves HPO-30**

To uncover these mechanisms, we examined known mutants displaying strong PVD dendrite defects. Previous studies showed that the proprotein convertase KPC-1 is required for PVD dendrite growth (Supplemental Data Fig. 1a, c, d) and that *kpc-1(null)* mutants have increased DMA-1 levels. However, it is unclear how furin-like KPC-1 regulates dendrite morphogenesis through DMA-1. To identify additional KPC-1 interactors that promote dendrite growth, we performed a genetic modifier screen on a weak *kpc-1* allele, *kpc-1(xr58)*. While *kpc-1(null)* mutants have dramatically reduced secondary branches and no quaternary branches, *kpc-1(xr58)* mutants have a mild reduction of secondary branches and dramatically reduced quaternary branches (Supplemental Data Fig. 3a, c, d). Our genetic modifier screen sought additional mutations that enhanced the dendrite phenotype of *kpc-1(xr58)*. From this screen, we identified *wy1008* (*hpo-30(E100K)*), which is a point mutation in HPO-30 that causes an E100K amino acid change in the first extracellular loop of HPO-30 (Supplemental Data Fig. 3b). *kpc-1(xr58); hpo-30(E100K)* double mutants showed a strong loss of dendrite similar to the *kpc-1(null)* mutants (Supplemental Data Fig. 3a-d). *hpo-30(E100K)* is likely a partial loss-of-function allele since the *hpo-30(E100K)* dendrite defects are milder than those of *hpo-30(null)* mutants, which generally fail to form quaternary branches but have longer secondary branches compared to the *kpc-1(null)* mutants (Supplemental Data Fig. 3b-d). Together, these data indicate a genetic interaction and possibly molecular interaction between *kpc-1* and *hpo-30* to regulate dendrite outgrowth.

Supplemental Figure 1

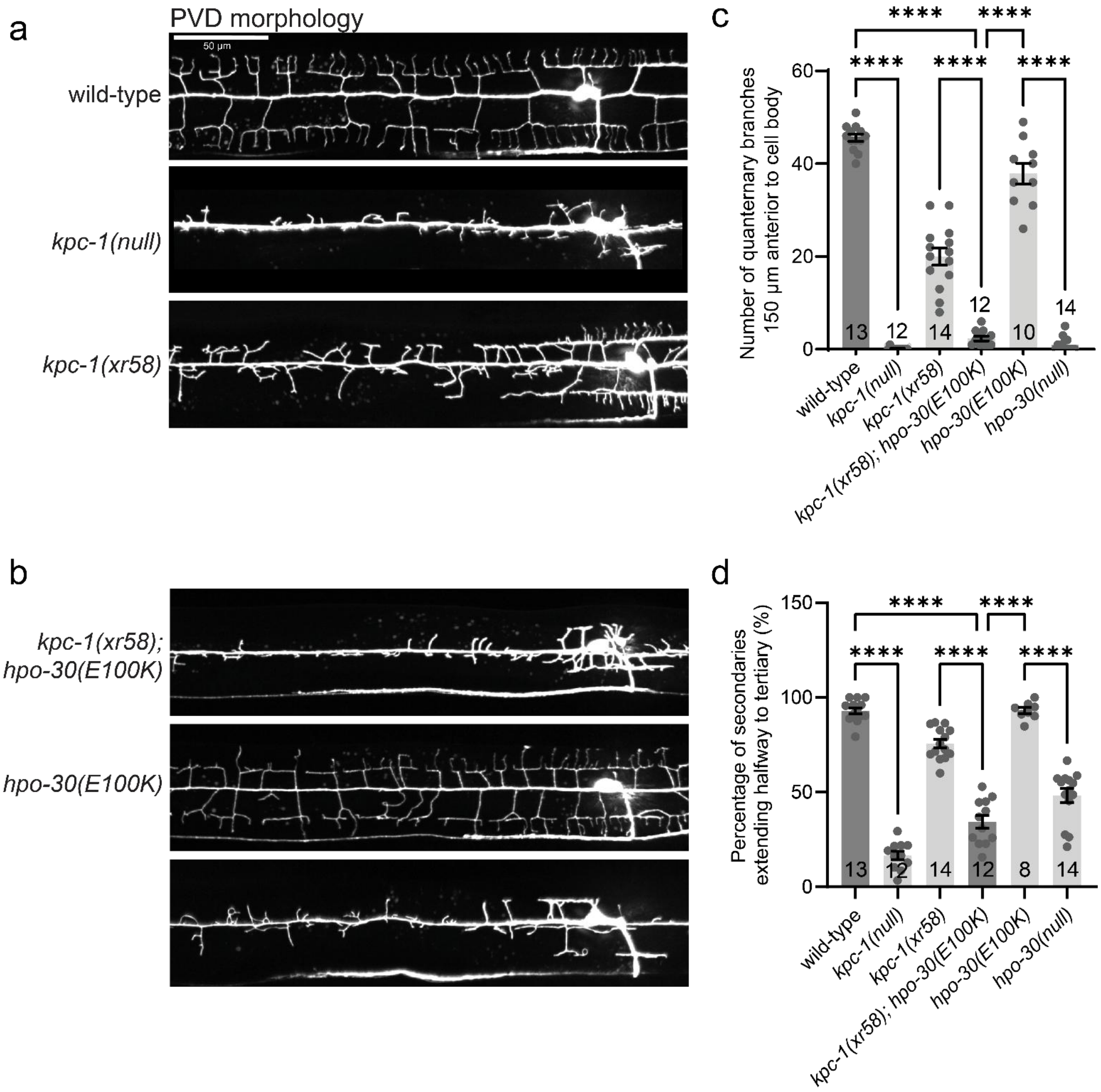


**Supplemental Data Figure 1. KPC-1 genetically interacts with HPO-30**

a, Representative images of PVD dendrite morphology in wild-type, *kpc-1(null),* and *kpc-1(xr58)* partial loss-of-function animals at the L4 stage. b, Representative images of PVD dendrite morphology in *kpc-1(xr58); hpo-30(wy1008)* double mutants, *hpo-30(wy1008)* single mutants, and *hpo-30(null)* animals at the L4 stage. c, Quantification of number of quaternary branches in a region 150 µm anterior to the PVD cell body, for genotypes shown in a and b. All P values, $P < 0.0001$. d, Quantification of percentage of extended secondaries for genotypes shown in a and b. All P values, $P < 0.0001$. Extended secondaries were measured as the percentage of secondary branches per worm that extended at least halfway to the tertiary dendrite line. For c, d, data are presented as mean values +/- SEM. n values within each bar. Statistical comparison was performed using Brown-Forsythe one-way ANOVA with Dunnett's multiple comparisons test. ****$P < 0.0001$.

**Tables**

**Table S1. Worm strains used in this study.**

| Strain Name | Genotype | Description |
|---|---|---|
| TV15911 | *wyIs592 III* | *ser2prom3::myr-gfp; Podr-1::rfp* |
| TV17248 | *sax-7(nj48) IV; wyIs592 III*[3] | Referred to as *sax-7(null)* |
| TV29097 | *dma-1(wy1924) I; wySi919 V* | *wy1924 = dma-1(ΔLRR), wySi919 = Pdes-2::myr-mScarlet::let-858* |
| TV19646 | *lect-2(ok2617) II; wyIs592 III* | *ok2617 = lect-2(null)* |
| TV14185 | wyEx5781; *sax-7(nj48)* | *wyEx5781 = pmec-17::SAX-7S::YFP(pXD38, 1.5ng/ul)+pmec-17::mnr-1(pXD86, 1.5ng/ul)+ser2prom3::myr-mCh(pOL036, 12ng/ul)*[27] |
| TV24863 | *hpo-30(zac227) V; wyIs592 III* | *zac227 = hpo-30(ΔICD)* |
| TV29380 | *hpo-30(zac227) V; dma-1(wy1924) I; wySi919 V* | |
| TV29319 | *hpo-30(zac227) V; dma-1(wy908) I; wyIs592 III* | *wy908 = dma-1(ΔICD)* |
| TV17465 | *dma-1 (wy908) I; wyIs592 III* | |
| TV29856 | *kpc-1(gk8) I; wyIs592 III* | Referred to as *kpc-1(null)* |
| TV17268 | *kpc-1(xr58) I; wyIs592 IIII* | |
| TV19400 | *kpc-1(xr58) I; hpo-30(wy1008) V; wyIs592 III* | *wy1008* = HPO-30(E100K) |
| TV19079 | *hpo-30(wy1008) V; wyIs592 III* | |

| | | |
|---|---|---|
| TV16821 | *hpo-30(ok2047) V; wyIs592 III* | Referred to as *hpo-30(null)* |
| TV24410 | *hpo-30(wy1220) V; wyIs592 III* | *wy1220 = hpo-30(R186A)* |
| TV24913 | *dma-1(wy1246) I* | wy1246 = *dma-1::GFP* (Endogenously tagged) |
| TV27036 | *dma-1(wy1246) I; kpc-1(gk8) I; wyIs581* | *wyIs581 = ser2prom3::myr-mCherry + Podr-1::gfp* |
| TV27089 | *dma-1(wy1246) I; hpo-30(wy1220) V* | |
| TV27759 | *dma-1(wy1246) I; rab-10(wy1616) I; wyIs910 X* | *wyIs910 =ser2prom3::FLP + Punc-122::bfp*<br>*wy1616* = flp-on mScarlet::RAB-10 |
| TV27781 | *dma-1(wy1246) I; rab-10(wy1616) I; kpc-1(gk8) I; wyIs910 X* | |
| TV27863 | *dma-1(wy1246) I; rab-10(wy1616) I; hpo-30(wy1220) V; wyIs910 X* | |
| TV27037 | *dma-1(wy1246) I; sax-7(nj48) IV* | |
| TV26091 | *dma-1(wy1437) I* | wy1437 = *dma-1(C470Y)::GFP* (Endogenously tagged) |
| TV27649 | *dma-1(wy1246) I; rab-10(wy1616) I; sax-7(nj48) IV; wyIs910 X* | |
| TV27865 | *dma-1(wy1437) I; rab-10(wy1616) I; wyIs910 X* | |
| TV26828 | *dma-1(zac98) I; wyIs592 III* | *zac98* = DMA-1(C470Y) |

| | | |
|---|---|---|
| TV17247 | *dma-1(tm5159) I; wyIs592 III* | Referred to as *dma-1(null)* |
| TV25655 | *hpo-30(wy1220) V; wyIs738; wyIs592 III* | *wyIs738 = ser2prom3::dma-1:gfp + Podr-1::gfp* |
| TV21180 | *hpo-30(ok2047) V; wyIs738; wyIs592 III* | |
| TV23577 | *wyEx9544; hpo-30(ok2047) V; wyIs592 III* | *wyEx9544 = ser2prom3::hpo-30(R186A)::gfp; Pmyo-2::mCherry* (Line 1) |
| TV23578 | *wyEx9545; hpo-30(ok2047) V; wyIs592 III* | *wyEx9545 = ser2prom3::hpo-30(R186A)::gfp; Pmyo-2::mCherry* (Line 2) |
| TV25343 | *wyEx10069; hpo-30(ok2047) V; wyIs587* | *wyEx10069 = ser2prom3::hpo-30:gfp; Pmyo-2::mCherry* |
| TV25344 | *wyEx10070; hpo-30(ok2047) V; wyIs587* | *wyEx10070 = ser2prom3::hpo-30(R186A)::gfp; Pmyo-2::mCherry* |
| TV27592 | *dma-1(wy1437) I; hpo-30(ok2047) V* | |
| TV27373 | *dma-1(wy1437) I; sax-7(nj48) IV* | |
| TV18815 | *wyEx7786; qyIs369* | *wyEx7786 = ser2prom3::hpo-30cDNA::mCherry; Podr-1::gfp*<br>*qyIs369 = ser2prom3::dma-1::gfp; unc-119 (+)* |
| TV27864 | *dma-1(wy1246) I; rab-10(wy1616) I; hpo-30(ok2047) V; wyIs910 X* | |
| TV27065 | *rab-10(wy1298) I; wyIs581 IV; wyIs910 X*[21] | *wy1298 = FRT::GFP::FRT::rab-10*<br>This strain uses the FLP-on system to cell-specifically tag RAB-10 in the PVD. |

| | | |
|---|---|---|
| TV29956 | *wyEx10802* | *wyEx10802 = ser2prom3::GFP::ced-10gDNA::unc-54 3'UTR; ser2prom3::mCherry::wormPAK-2(binding domain)::mCherry; Podr-1::GFP* |
| TV30055 | *wyEx10802; dma-1 (tm5159) I* | Referred to as *dma-1(null)* |
| TV30056 | *wyEx10802; hpo-30(wy1220) V* | *wy1220 = hpo-30(R186A)* |
| TV30057 | *wyEx10802; dma-1 (wy1924) I* | *wy1924 = dma-1(ΔLRR)* |
| TV28583 | *dma-1(wy1286); hpo-30(qyIs366)* | wy1286 = *dma-1::tagRFP*<br>*qyIs366 = ser2prom3::hpo-30::gfp; unc-119 (+)* |

**Table S2. S2 cell plasmids used in this study.**

| Plasmid Name | Description |
|---|---|
| pXD224 | *Pactin::hpo-30:gfp* |
| pRDS044 | *Pactin::FLAG:hpo-30* |
| pML001 | *Pactin::hpo-30(R40A)::gfp* |
| pML002 | *Pactin::hpo-30(R71A)::gfp* |
| pML003 | *Pactin::hpo-30(R74A)::gfp* |
| pML004 | *Pactin::hpo-30(R88A)::gfp* |
| pML005 | *Pactin::hpo-30(R99A)::gfp* |
| pML006 | *Pactin::hpo-30(R105A)::gfp* |
| pML007 | *Pactin::hpo-30(R186A)::gfp* |
| pML008 | *Pactin::hpo-30(R187A)::gfp* |
| pML009 | *Pactin::hpo-30(R189A)::gfp* |
| pML010 | *Pactin::hpo-30(R198A)::gfp* |
| pRDS032 | *Pactin::hpo-30(E100K)::gfp* |
| pXD54 | *Pactin::dma-1::rfp* |
| pXD49 | *Pactin::sax-7s::gfp* |
| pXD85 | *Pactin::mnr-1::gfp* |

| | |
|---|---|
| pWZ263 | *Pactin::lect-2::3xFLAG* |
| pRDS077 | *Pactin::dma-1(C470Y)::rfp* |
| pXYH9 | *ser2prom3::gfp::ced-10* |
| pXYH5 | *ser2prom3::mCherry::wormPAK-2(binding domain)::mCherry* |
