# Supplementary material for: Stochastic growth and ligand–receptor interaction-mediated stabilization generate stereotyped dendritic arbors
Source: Nat Neurosci. 2026 May 4;29(6):1313–26. doi: 10.1038/s41593-026-02278-0 (PMC13246438; doi:10.1038/s41593-026-02278-0)
Supplement: Supplementary file 2 — Reporting Summary [file 41593_2026_2278_MOESM2_ESM.pdf]

nature portfolio

Corresponding author(s): Kang Shen

Last updated by author(s): Feb 9, 2026

Reporting Summary

Nature Portfolio wishes to improve the reproducibility of the work that we publish. This form provides structure for consistency and transparency in reporting. For further information on Nature Portfolio policies, see our Editorial Policies and the Editorial Policy Checklist.

Statistics

For all statistical analyses, confirm that the following items are present in the figure legend, table legend, main text, or Methods section.

n/a	Confirmed	
☐	☒	The exact sample size (*n*) for each experimental group/condition, given as a discrete number and unit of measurement
☐	☒	A statement on whether measurements were taken from distinct samples or whether the same sample was measured repeatedly
☐	☒	The statistical test(s) used AND whether they are one- or two-sided<br>*Only common tests should be described solely by name; describe more complex techniques in the Methods section.*
☐	☒	A description of all covariates tested
☐	☒	A description of any assumptions or corrections, such as tests of normality and adjustment for multiple comparisons
☐	☒	A full description of the statistical parameters including central tendency (e.g. means) or other basic estimates (e.g. regression coefficient) AND variation (e.g. standard deviation) or associated estimates of uncertainty (e.g. confidence intervals)
☐	☒	For null hypothesis testing, the test statistic (e.g. *F*, *t*, *r*) with confidence intervals, effect sizes, degrees of freedom and *P* value noted<br>*Give P values as exact values whenever suitable.*
☒	☐	For Bayesian analysis, information on the choice of priors and Markov chain Monte Carlo settings
☒	☐	For hierarchical and complex designs, identification of the appropriate level for tests and full reporting of outcomes
☒	☐	Estimates of effect sizes (e.g. Cohen's *d*, Pearson's *r*), indicating how they were calculated

Our web collection on statistics for biologists contains articles on many of the points above.

Software and code

Policy information about availability of computer code

Data collection	Fiji(Image J) Version 1.54p
Data analysis	Graphpad Prism 10 version 10.5.0

For manuscripts utilizing custom algorithms or software that are central to the research but not yet described in published literature, software must be made available to editors and reviewers. We strongly encourage code deposition in a community repository (e.g. GitHub). See the Nature Portfolio guidelines for submitting code & software for further information.

Data

Policy information about availability of data

All manuscripts must include a data availability statement. This statement should provide the following information, where applicable:

- Accession codes, unique identifiers, or web links for publicly available datasets
- A description of any restrictions on data availability
- For clinical datasets or third party data, please ensure that the statement adheres to our policy

All data generated or analyzed during this study are included in the manuscript and supporting files, source data files have been provided. Strains and plasmids given upon request. All data needed to evaluate the conclusions in the paper are present in the paper and/or the Supplementary Materials and will be made available.

Research involving human participants, their data, or biological material

Policy information about studies with human participants or human data. See also policy information about sex, gender (identity/presentation), and sexual orientation and race, ethnicity and racism.

Reporting on sex and gender	N/A
Reporting on race, ethnicity, or other socially relevant groupings	N/A
Population characteristics	N/A
Recruitment	N/A
Ethics oversight	N/A

Note that full information on the approval of the study protocol must also be provided in the manuscript.

Field-specific reporting

Please select the one below that is the best fit for your research. If you are not sure, read the appropriate sections before making your selection.

☒ Life sciences ☐ Behavioural & social sciences ☐ Ecological, evolutionary & environmental sciences

For a reference copy of the document with all sections, see nature.com/documents/nr-reporting-summary-flat.pdf

Life sciences study design

All studies must disclose on these points even when the disclosure is negative.

Sample size	No sample sizes calculation was performed. No statistical methods were used to pre-determine sample sizes but our sample sizes are similar to those reported in previous publications (Tao 2019, Shi 2021).
Data exclusions	No data were excluded for analysis.
Replication	For key results, experiments include multiple technical repeats, conducted on different days and replicated by different individuals to ensure reproducibility. All attempts at replication were successful.
Randomization	Animals that were picked for experiments are random within a given genotype.
Blinding	FLIM experiments were conducted blindly, but PVD morphology experiments and western blots were not because many of the mutants used could be easily identified by their morphology during imaging. However, the same experiments were conducted and replicated by multiple different researchers and they were reproducible.

Reporting for specific materials, systems and methods

We require information from authors about some types of materials, experimental systems and methods used in many studies. Here, indicate whether each material, system or method listed is relevant to your study. If you are not sure if a list item applies to your research, read the appropriate section before selecting a response.

Materials & experimental systems

n/a	Involved in the study	
☐	☒	Antibodies
☐	☒	Eukaryotic cell lines
☒	☐	Palaeontology and archaeology
☐	☒	Animals and other organisms
☒	☐	Clinical data
☒	☐	Dual use research of concern
☒	☐	Plants

Methods

n/a	Involved in the study	
☒	☐	ChIP-seq
☒	☐	Flow cytometry
☒	☐	MRI-based neuroimaging

Antibodies

Antibodies used	mouse Anti-GFP and mouse anti-FLAG. mouse antibody to GFP (1:2000, Roche #11814460001), mouse antibody to FLAG (1:1000, Sigma F3165)
Validation	Validation of mouse anti-gfp and mouse anti-flag were validated in fig. 4

Eukaryotic cell lines

Policy information about cell lines and Sex and Gender in Research

Cell line source(s)	Drosophila S2 cells
Authentication	None of the cells were authenticated.
Mycoplasma contamination	Cell lines were not tested for Mycoplasma contamination.
Commonly misidentified lines (See ICLAC register)	No commonly misidentified cell lines were used in this study.

Animals and other research organisms

Policy information about studies involving animals; ARRIVE guidelines recommended for reporting animal research, and Sex and Gender in Research

Laboratory animals	C. elegans were used.
Wild animals	No wild animals were used.
Reporting on sex	Only hermaphrodites were used.
Field-collected samples	Done of the samples were collected in the field.
Ethics oversight	Ethnics not required for c. elegans work. The only animals used in this study are C. elegans nematodes, which are not subject to animal welfare regulations. No ethical approval was required.

Note that full information on the approval of the study protocol must also be provided in the manuscript.
