# Supplementary material for: Stochastic growth and ligand–receptor interaction-mediated stabilization generate stereotyped dendritic arbors
Source: Nat Neurosci. 2026 May 4;29(6):1313–26. doi: 10.1038/s41593-026-02278-0 (PMC13246438; doi:10.1038/s41593-026-02278-0)

Uncropped blots

Blot for Fig. 4a

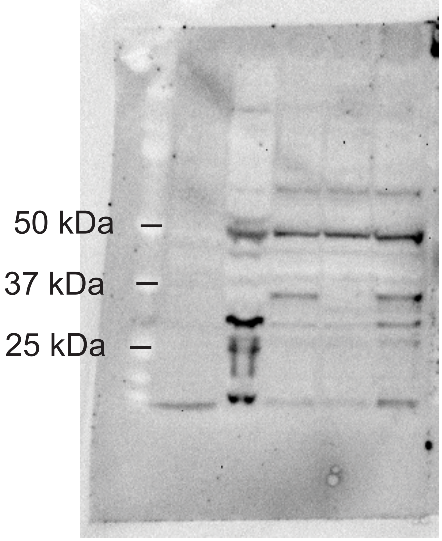

Blot for Fig. 4b (left)

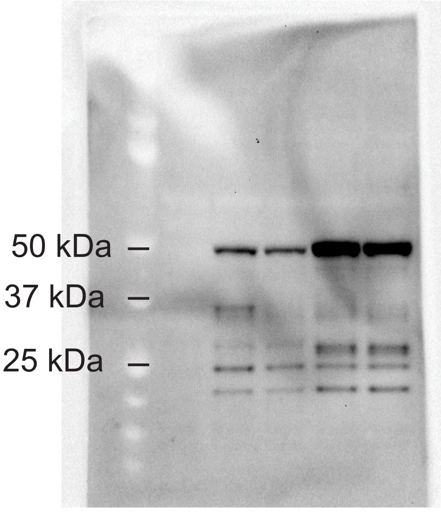

Blot for Fig. 4b (right)

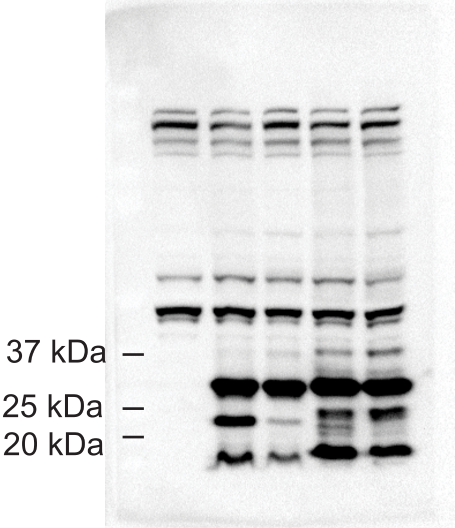

Blot for Extended Data Fig. 4a

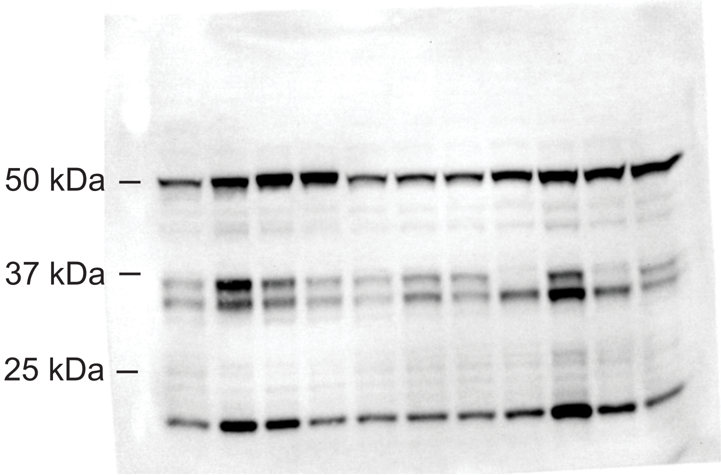

Blot for Extended Data Fig. 4b - input and DMA-1::HA

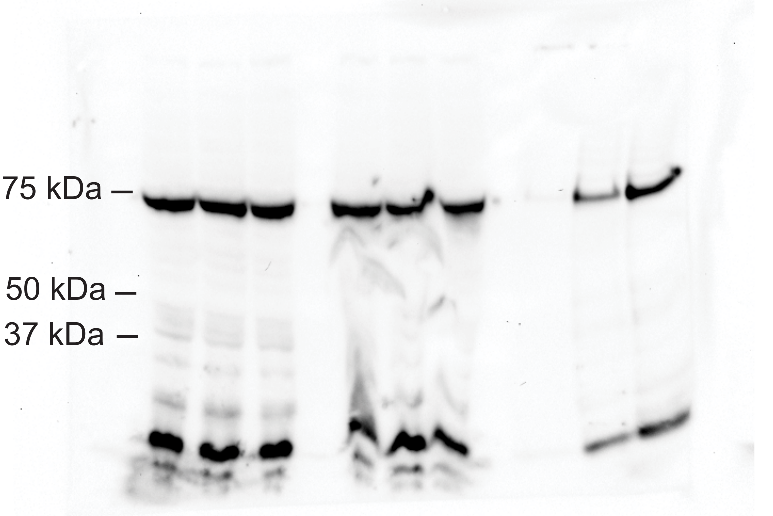

Blot for Extended Data Fig. 4b - HPO-30::GFP

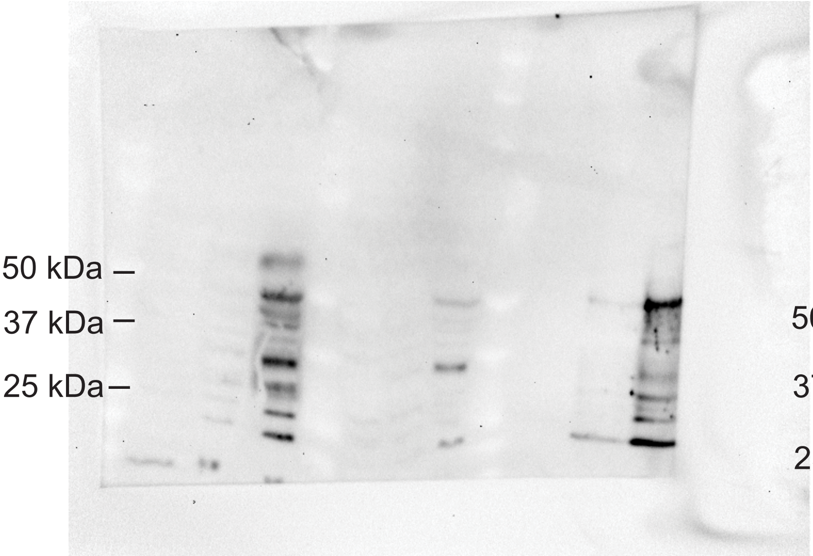

Blot for Extended Data Fig. 5j

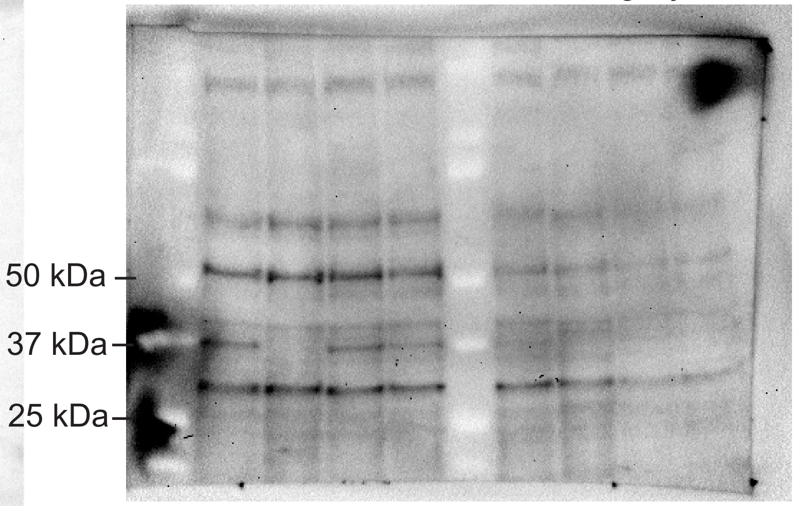

Supplement: Supplementary file 14 — Unprocessed western blots. [file 41593_2026_2278_MOESM14_ESM.pdf]
